# Antidiabetic Phytochemicals From Medicinal Plants: Prospective Candidates for New Drug Discovery and Development

**DOI:** 10.3389/fendo.2022.800714

**Published:** 2022-02-24

**Authors:** Safaet Alam, Md. Moklesur Rahman Sarker, Taposhi Nahid Sultana, Md. Nafees Rahman Chowdhury, Mohammad A. Rashid, Nusrat Islam Chaity, Chao Zhao, Jianbo Xiao, Elsayed E. Hafez, Shah Alam Khan, Isa Naina Mohamed

**Affiliations:** ^1^ Department of Pharmacy, State University of Bangladesh, Dhaka, Bangladesh; ^2^ Pharmacology and Toxicology Research Division, Health Med Science Research Limited, Dhaka, Bangladesh; ^3^ Department of Pharmacy, University of Asia Pacific, Dhaka, Bangladesh; ^4^ Department of Pharmacy, University of Dhaka, Dhaka, Bangladesh; ^5^ Department of Pharmaceutical Chemistry, Faculty of Pharmacy, University of Dhaka, Dhaka, Bangladesh; ^6^ College of Food Science, Fujian Agriculture and Forestry University, Fuzhou, China; ^7^ Department of Analytical Chemistry and Food Science, Faculty of Food Science and Technology, University of Vigo, Vigo, Spain; ^8^ Plant Protection and Biomolecular Diagnosis Department, ALCRI (Arid Lands Cultivation Research Institute), City of Scientific Research and Technological Applications, Alexandria, Egypt; ^9^ College of Pharmacy, National University of Science & Technology, Muscat, Oman; ^10^ Pharmacology Department, Medicine Faculty, Universiti Kebangsaan Malaysia (The National University of Malaysia), Kuala Lumpur, Malaysia

**Keywords:** diabetes mellitus, antidiabetic, antihyperglycemic, phytochemical, phytomedicine, bioactive compound, drug discovery, drug development

## Abstract

Diabetes, a chronic physiological dysfunction affecting people of different age groups and severely impairs the harmony of peoples’ normal life worldwide. Despite the availability of insulin preparations and several synthetic oral antidiabetic drugs, there is a crucial need for the discovery and development of novel antidiabetic drugs because of the development of resistance and side effects of those drugs in long-term use. On the contrary, plants or herbal sources are getting popular day by day to the scientists, researchers, and pharmaceutical companies all over the world to search for potential bioactive compound(s) for the discovery and development of targeted novel antidiabetic drugs that may control diabetes with the least unwanted effects of conventional antidiabetic drugs. In this review, we have presented the prospective candidates comprised of either isolated phytochemical(s) and/or extract(s) containing bioactive phytoconstituents which have been reported in several *in vitro*, *in vivo*, and clinical studies possessing noteworthy antidiabetic potential. The mode of actions, attributed to antidiabetic activities of the reported phytochemicals and/or plant extracts have also been described to focus on the prospective phytochemicals and phytosources for further studies in the discovery and development of novel antidiabetic therapeutics.

## Introduction

Diabetes mellitus is a type of chronic metabolic disorder categorized by insufficiency in insulin activity and/or insulin secretion. Anomalies in proteins, carbohydrates and lipids metabolism can arise due to the lack of insulin, an anabolic hormone (1). These abnormalities in metabolism are caused by low levels of insulin, insulin resistance of target tissues, insulin receptor level, primarily skeletal muscles, and adipose tissue and to a lesser degree, liver, signal transduction system, and/or effector enzymes or genes and/or signal transduction pathway (2). Diabetes is one of the most abundant metabolic diseases across the world accounting for about 2.8% of the population worldwide and is projected to reach 4.4% by 2030 which has already risen to an unprecedented extent of the epidemic (3). Despite being a non-communicable disorder, diabetes is considered one of the five biggest morbidities worldwide (1). Diabetes category and frequency vary depending on the severity of the symptoms. Some patients with diabetes are asymptomatic, particularly patients with type 2 diabetes during the initial periods of illness whereas others have noticeable hyperglycemia. Uncontrolled and unmonitored diabetes can lead to stupor, coma and even death if kept untreated because of ketoacidosis or rare non-ketotic hyperosmolar disorders (4). The development of diabetes may include the interaction of genetic and non-genetic factors (5). Despite diabetes classification being crucial and having repercussions for treatment policies, this is somehow ambiguous and many diabetic individuals do not easily accommodate into one class, exclusively younger adults and 10% of the initially classified patients can need revision afterward (6). The standard classification of diabetes as type 1, type 2 and gestational diabetes mellitus (GDM) as introduced by the American Diabetes Association (ADA) in 1997 remains the best-accepted and adopted by ADA (4).

**Figure 1:**
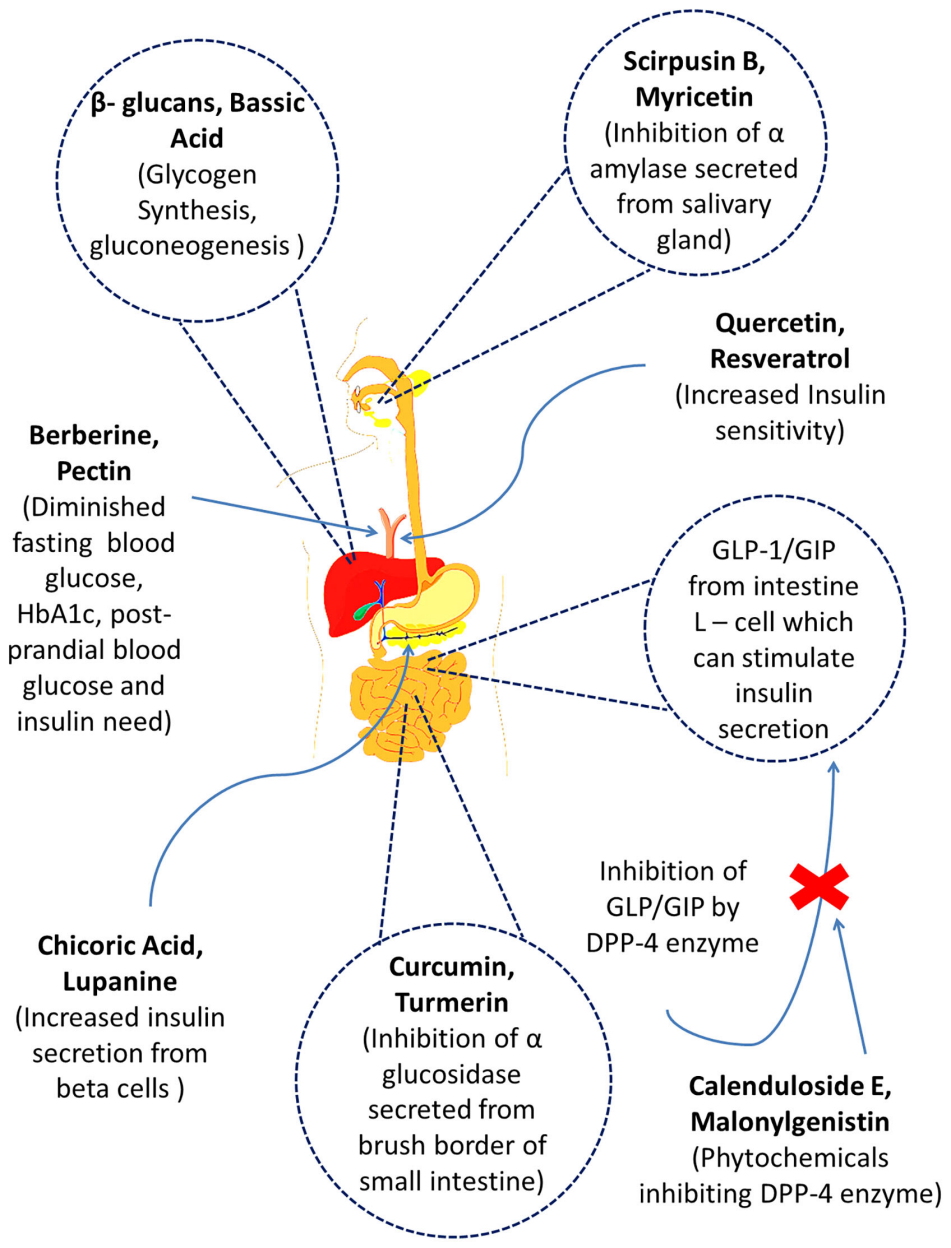
The mechanisms of action of several prospective bioactive secondary metabolites (phytochemicals) obtained from different medicinal plants.

**Figure 2:**
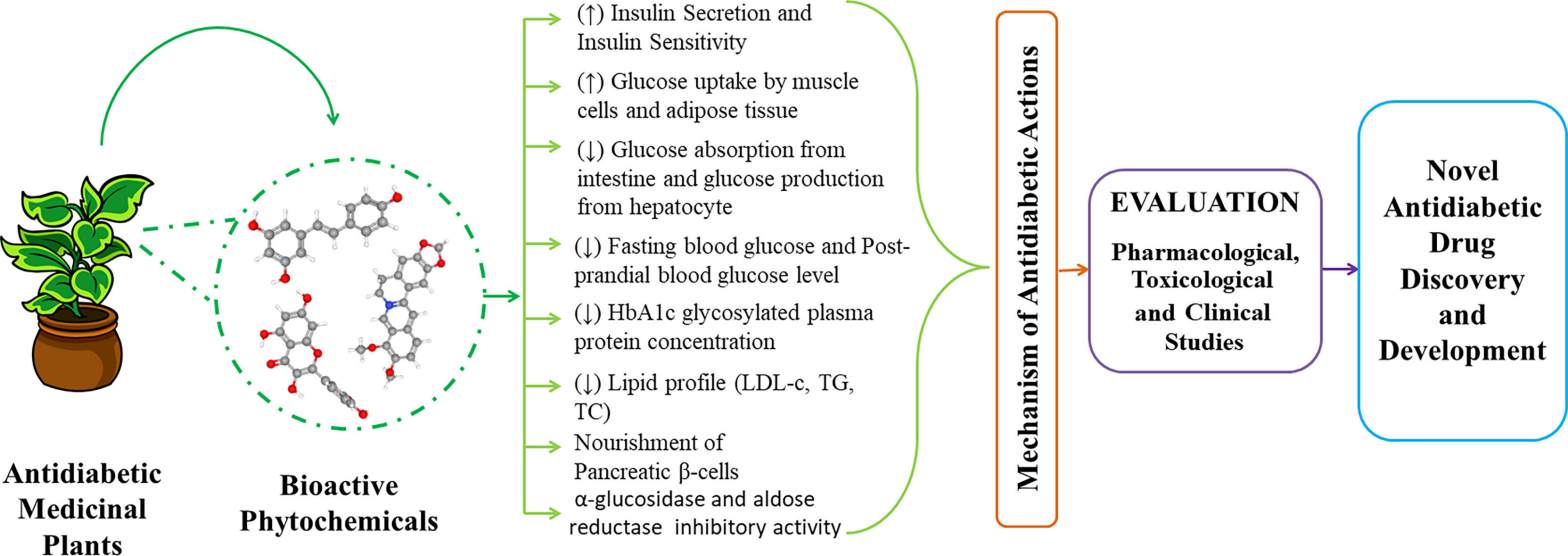
Graphical abstract of prospective antidiabetic phytochemicals from medicinal plants for the discovery and development.

Currently, there are many antidiabetic drugs available in the market to treat hyperglycemia which notably works *via* improvement of insulin sensitivity, complementing insulin, upraising insulin secretion and stimulating glucose uptake. But metformin and sulfonylureas type antidiabetic drugs are compromised with several unwanted side effects such as diarrhea and lactic acidosis (demonstrated by metformin) and hepatic failure, weight gain, tachycardia and hypothyroidism (demonstrated by sulfonylureas) (7). Plant is always considered as one of the most reliable sources of curing agents of diseases and many of those synthetic drugs are either directly or indirectly derived from them. Plants and plant products can exert promising antidiabetic efficacy based on recent studies (**Figure 1**). Plant sources of antidiabetic agents are very much popular from the ancient era as they are relatively safer and much cheaper alternatives than synthetic drugs and are also mentioned in many folkloric medicines including the Indian, Korean and Chinese culture. Traditional herbal medicines and functional foods are believed to ameliorate diabetic syndromes *via* six notable mechanism of actions including enhanced insulin secretion and sensitivity, glucose uptake by muscle cells and adipose tissues and inhibition of glucose absorption from intestine and glucose production from hepatocytes along with demonstrating anti-inflammatory properties (7). As a result, functional foods and phytotherapies are becoming popular across the world day by day (8). In the current review, we have compiled most notable medicinal and dietary plants along with their isolated antidiabetic phytochemicals to give distinct insights into the establishment of novel functional foods and drug moieties against diabetes. The graphical abstract of the manuscript has been presented in **Figure 2**.

## Latest Researches on the Molecular Mechanisms and Pathogenies of Diabetes Mellitus

In type 1 Diabetes, the pancreatic β-cells undergo autoimmune destruction by CD4+ and CD8+ T cells and macrophages which result in insulin deficiency (9, 10). Islet cell antibodies are found in nearly 85% of the patients, and most of them act against the glutamic acid decarboxylase (GAD) found inside the β-cells of the pancreas (9).

The metabolic disorders related to type 1 diabetes mellitus are a result of deficiency of insulin secretion caused by the immune destruction of islets of Langerhans of the pancreas. Besides, pancreatic α-cells start to function abnormally and secrete an excessively large amount of glucagon in patients with type 1 diabetes mellitus, which further aggravates the metabolic disorders already caused by insulin deficiency (11). A deficiency of insulin causes lipolysis to occur at an uncontrolled rate which causes the amount of free fatty acids in the blood to rise resulting in a reduction of glucose metabolism in the peripheral tissues (11). The deficit of insulin also causes a reduction of glucokinase enzyme in the liver and the GLUT-4 transporter protein in adipose tissue resulting in an inability of the target tissues to respond normally to insulin.

Impaired secretion of insulin through destruction of the insulin secreting β-cells, and diminished insulin activity through insulin resistance marks the underlying mechanisms of the pathogenesis of type 2 diabetes (9). The mitochondria- endoplasmic reticulum contacts are known as mitochondria-associated membranes (MAMs) play an important role in the regulation of lipid exchange, signaling of calcium, cell survival, and homeostasis in cellular metabolism. These MAM contacts are known to contain several insulin signaling proteins such as AKT kinase, mTORC2, PP2A, and PTEN and thus participate in insulin signaling. A growing number of studies have shown that these MAMs are involved in causing dysfunction of the insulin producing β cells, resistance to insulin in the peripheral tissues, leading to type 2 diabetes mellitus (12). miRNAs are small RNA consisting of 20–24 nucleotides that regulates early development, fat metabolism, cell proliferation, differentiation, apoptosis, and death. Recent studies have shown that these miRNAs contribute to the pathogenesis of type 2 diabetes mellitus and may be developed into new biomarkers (13).

As reactive oxygen species impact chemical changes in all cellular components and produce lipid peroxidation, oxidative stress also causes type 2 diabetes mellitus. As a result, lipid peroxidation is another important cause of type 2 diabetes mellitus (14). In excess amounts of hydrogen peroxide (H_2_O_2_), DNA, RNA, and lipids are severely damaged. Catalase (CAT) is the major H_2_O_2_ regulator, and it neutralizes H2O2 by catalytically converting it to water and oxygen. When catalases (CAT) are deficient, pancreatic islet-cells are more susceptible to excessive formation of reactive oxygen species (ROS) and oxidative stress, which leads to pancreatic islet dysfunction and overt type 2 diabetes mellitus (15). In numerous illness states involving oxidative stress as a significant causal factor, such as diabetes and obesity, plasma levels of oxidized low-density lipoprotein (oxLDL) are elevated (16). Nuclear factor kappa B (NF-B), NH2-terminal Jun kinases, and p53 MAPK are transcriptionally regulated pathways that have been considered one of the most important stress-signaling pathways, and oxidative stress plays a role in the development of type 2 diabetes mellitus through their involvement (14).

Type 2 diabetes mellitus is linked by decreased physical activity and exercise training, as well as increased sedentary habits, which are linked to elevated indicators of chronic systemic inflammation (17). Proinflammatory molecules such as interleukin 6 (IL-6), C-Reactive Protein (CRP), tumor necrosis factor-alpha (TNF-) and interleukin 1 (IL-1) are released into the bloodstream and inside specific organs in this scenario, causing metabolic inflammation (18). IL-1 is involved in the pancreatic autoimmune response, decreasing -cell activity and activating the nuclear factor kappa-light-chain-enhancer of activated B cells (NF-B) transcription factor, inhibiting -cell function and inducing death.

The importance of the gut microbiota in the development of diabetes has been demonstrated, and new studies suggest that dysbiosis can increase type 2 diabetes mellitus (19). Experiments in animal models showed that a high-fat diet can increase the synthesis of lipopolysaccharide (from Gram-negative bacteria) by up to thrice, contributing to low-grade inflammation and insulin resistance (20, 21). Intestinal dysbiosis can also impair short-chain fatty acid production, which is important for gut barrier integrity, pancreatic cell proliferation, and insulin biosynthesis (22, 23). Dysbiosis can also affect the production of other metabolites such as branched aminoacids and trimethylamine, causing glucose homeostasis to be disrupted and type 2 diabetes mellitus to develop (24, 25). The clinical consequences of the gut microbiome are still being researched, and more study is needed to better understand the link between gut bacteria and type 2 diabetes mellitus (26).

## Articles Search Strategy

An extensive literature search was carried out through the following databases: Web of Science, Scopus, PubMed/Medline, ScienceDirect, ClinicalTrials.gov, Wiley Online Library and Google Scholar. The following keywords were used: ‘Antidiabetic’, ‘Diabetes’, ‘Phytochemical’, ‘Bioactive compound’, ‘Type 2 diabetes’, ‘Pharmacology’, and ‘Clinical trial’. Only peer-reviewed scientific journals were considered during the process. Plants with reported antidiabetic phytochemicals along with mechanism of actions have been considered only for the review work. Of the 598 identified papers and clinical trials records, 295 unique articles were included and reported in this comprehensive review following inclusion criteria.

## *Anoectochilus roxburghii* (Wall.) Lindl.

*Anoectochilus roxburghii* (Wall.) Lindl. (family: Orchidaceae) is a perennial herb which mainly occurs in China, Taiwan, Japan, Sri Lanka, India, and Nepal. Polysaccharides from *A. roxburghii* lower blood glucose levels by enhancing the body’s antioxidant capacity, reducing blood lipid levels, modulating the activity of glucose-metabolizing enzymes, minimizing tissue damage such as pancreas, and promoting damaged tissue repair (27). Kinsenoside, extracted from *A. roxburghii* was found to demonstrate significant hypoglycemic activity. Previous studies have shown that kinsenoside could help in the restoration of damaged β cells in pancreas and function against oxidative stress and NO factor and also regulates antioxidant enzymes, scavenging of free radicals (28).

## *Bacopa monnieri* (L.) Wettst.

*Bacopa monnieri* (L.) Wettst. (family: Scrophulariaceae) is a creeping herb which occurs across India. Several compounds including tetracyclic triterpenoid saponins, Bacosides A and B, Hersaponin, alkaloids viz. Herpestine and Brahmine and flavonoids have been isolated from the plant (29). Bacosine, a triterpene isolated from the ethyl acetate fraction of the ethanolic extract of *B. monnieri* showed pronounced reduction in blood glucose levels in diabetic rats in a dose-dependent mode; however, no such effect has been observed on normal rats. Thus, bacosine is known to possess antihyperglycemic properties rather than hypoglycemic activity. It has been suggested that bacosine works in a way similar to insulin and that its antihyperglycemic activity might be attributed to the increase in the consumption of peripheral glucose as well as protect against oxidative damage in alloxan induced diabetes. Bacosine caused a pronounced increase (p < 0.001) of glycogen content in the liver of diabetic rats and consequently proved its insulin-like activity resulting in an increased uptake of glucose (29).

## *Berberis aristata* DC.

*Berberis aristata* DC (family: Berberidaceae) is native to Northern Himalayan part and is known locally as ‘Daruhaldi’ or ‘Citra’. Root extract *B. aristata* regulates glucose homeostasis by reducing gluconeogenesis and oxidative stress and exhibits a strong anti-hyperglycemic activity (30, 31). The major antidiabetic compound extracted from this plant is berberine. Berberine is known to act through several mechanisms, including insulin-mimetic activity; improving the action of insulin by triggering AMPK (5′ adenosine monophosphate-activated protein kinase); reducing insulin resistance through protein kinase C-dependent up-regulation of insulin receptor expression; causing glycolysis; and by enhancing GLP-1 (Glucagon-like peptide 1) secretion and regulating its release, and by inhibiting DPP-IV (Dipeptidyl peptidase-4) (30). According to 32, berberine extracted from *B. aristata* in a manner of 0.5 gm thrice a day on type 2 diabetic patients showed equal efficacy to metformin monotherapy in the reduction of fasting blood glucose, HbA1c, postprandial blood glucose, postprandial insulin and basal insulin (32). Another clinical trial also showed the efficacy of berberine as adjuvant therapy in poorly controlled type 2 diabetic patients (32). In other clinical trials, berberine also showed promising efficacy compared to rosiglitazone and metformin by reducing HbA1c, fasting blood glucose, postprandial blood glucose despite having some adverse effects on liver (33). In another 6 months long randomized-controlled trial on 85 type 1 diabetic patients (39 males and 46 females), twice daily intake of a tablet containing 588/105 mg combination of *Berberis aristata/Silybum marianum* decreased insulin use by the body during insulin therapy. Moreover, there was a decrease in Hb1Ac level in comparison to baseline and in fasting and postprandial plasma glucose levels in comparison to both baseline and placebo (34). This combination, which is made to improve the low bioavailability of berberine in oral route, has also been reported to show notable antidiabetic effects in 136 obese/overweight type 2 diabetic patients, as per another year-long placebo-controlled study (35).

## *Bixa Orellana* L.

*Bixa Orellana* L. (family: Bixaceae) also known by the name ‘Achuete’, is a rapidly growing shrub that can grow upto 3 to 5 meters in height. The plant originated in Brazil but also grows in South and Central America. Besides, it also occurs in tropical countries such as Peru, Mexico, Ecuador, Indonesia, India, Kenya, and East Africa (36). *B. Orellana* lowered the blood glucose levels in dogs with streptozotocin-induced diabetes (36, 37). It has been suggested that *B. Orellana* causes a reduction in blood glucose level by increasing peripheral utilization of glucose (38, 39), increasing plasma insulin levels and increasing the binding of insulin to insulin receptors (40). Bixin, a high carotenoid content and a natural pigment found in *B. orellana* showed prominent hypoglycemic actions. Bixin is commercially named “Annatto” and a very promising wellspring of new medicines as well imperative nutraceuticals (36).

## *Bumelia sartorum* Mart.

*Bumelia sartorum* Mart. (family: Sapotaceae) is a large and tall tree commonly known as ‘Quixaba’, ‘Quixabeira’, ‘Tranceporteira’, ‘Sacutiaba’ and ‘Rompe-gibao’ in northeastern Brazil. It naturally grows from north of Minas Gerias to Piaui (41). An unsaturated triterpene acid named bassic acid, isolated from the ethanol extract of root bark of *B. surtorum* exhibited significant hypoglycemic activity in alloxan induced diabetic rat models. Moreover, bassic acid was found to significantly increase the glucose uptake and glycogen synthesis process in isolated rat diaphragm. In alloxan-diabetic rats following bassic acid treatment, a significant increase in plasma insulin levels was found. It has been suggested that bassic acid increases insulin secretion from pancreatic beta-cells. This could be the underlying mechanism by which bassic acid shows its hypoglycemic property which was found to be approximately equal to that of chlorpropamide (42).

## *Callistemon rigidus* R.Br.

*Callistemon rigidus* R.Br. (family: Myrtaceae) is an evergreen plant which is native to Australia. Noteworthy antidiabetic compounds piceatannol and scirpusin B were isolated from the stem bark of the plant using ^1^H- and ^13^C-NMR technology (43). These compounds can suppress the activity of α- amylase in isolated mouse plasma Methanol extract of *C. rigidus* can also demonstrate prominent repressing activity on α- amylase. Besides, scirpusin B can regulate α-amylase in mouse GIT to demonstrate antidiabetic efficacy. These compounds are also expected to abate increment of postprandial glucose level and can offer a very good wellspring of antidiabetic drug development (44).

## *Catharanthus roseus* (L.) G.Don

*Catharanthus roseus* (L.) G.Don (family: Apocynaceae) is a shrub-type plant that can grow up to 30–100 cm in height. The plant originated from Madagascar but is available around the world due to its high survival rate (45). Its leaf extracts exhibited significant dose-dependent blood sugar-lowering activity in normal and streptozotocin-induced diabetic model rats. The blood sugar-lowering potential of the leaf extract was equivalent to that of the commercially available drug Tolbutamide in the animal models (45, 46). Comapred to normal animals, the enzymic activities of glycogen synthase, glucose 6-phosphate-dehydrogenase, succinate dehydrogenase and malate dehydrogenase were found to decrease in the liver of diabetic animals and were significantly increased after treatment with dichloromethane–methanol (DCMM) extract of leaves and twigs of *C. roseus* at dose 500 mg/kg p.o. for 7 days. It has been suggested that *C. roseus* exhibits its anti-diabetic activity by increasing glucose metabolism in treated rats (47). Among isolated compounds, especially alkaloids including vindoline, vindolidine, vindolicine and vindolinine, isolated from *Catharanthus roseus* leaves induced increased glucose uptake in myoblast C2C12 or pancreatic β-TC6 cells where vindolicine showed maximum efficacy. First three compounds did not exhibit any cytotoxicity towards pancreatic β-TC6 cells even when administered in the maximum dose of 25.0µg/mL. Vindolicine, vindolidine and vindolinine also revealed improved protein tyrosine phosphatase-1B (PTP-1B) inhibition actions which can play a pivotal role in type 2 diabetes management (45).

## *Chamaemelum nobile* (L.) All.

*Chamaemelum nobile* (L.) All. (family: Asteraceae) is a native South-eastern Moroccan shrub-type which is locally known as ‘Babounge’. It is very much popular throughout Europe, most notably in France and USA (48). Its aqueous extract mitigated blood glucose concentration in streptozotocin induced diabetic rat models except changing plasma insulin level which indicated an insulin secretion independent pathway (48). *C. nobile* may also exhibit its hypoglycaemic activity in the gastrointestinal tract by slowing down the digestion process and reducing the rate of carbohydrate absorption (48). Chamaemeloside, 3-hydroxy-3-methylglutaric acid (HMG) containing flavonoid glucoside is the most notable isolated antidiabetic compound from this plant which revealed hypoglycemic activity in Swiss-Webster mice models by reducing plasma glucose concentration. The reduction of fasting glucose level and improved glucose tolerance referred that it might act following more than one mechanism (49). The underlying mechanism of action may be attributed to the stimulation of the utilization of peripheral, especially in muscle and adipose tissue. In 8 week long clinical study on 26 pre-diabetic volunteers (21 were male and 5 were female; mean age: 50.5 ± 8.5 years), a mixed herbal extract supplementation was made by combining hot water extract of *Anthemis nobilis* (Roman chamomile), which is a synonym of *Chamaemelum nobile* and *Vitis vinifera* in the dose of 1200 mg reportedly reduced abnormal glucose values and thus the risk of developing diabetes (50).

## *Cichorium intybus* L.

*Cichorium intybus* L. (family: Asteraceae) is an erect herb-type perennial plant that grows upto 1 m. It is abundantly found in Asia, Africa, Europe, and Southern America. It is well-known as ‘Chicory’ (51). Its ethanolic extract (CIE) was found to show a marked reduction in the hepatic glucose-6-phosphatase (Glc-6-Pase) activity when compared to the control group. The decrease in the activity of hepatic Glc-6-Pase could lead to a reduction in the production of hepatic glucose, which in turn reduces the blood glucose level in CIE-treated diabetic rats (52). Chlorogenic acid and chicoric acid are the two most notable antidiabetic phytoconstituents isolated from this plant which can increase glucose uptake in L6 muscular cells. Both phytochemicals can also upraise insulin secretion from the INS-1E insulin-secreting cell line and rat islets of Langerhans. Besides, chicoric acid can exert both insulin-secreting and sensitizing activities (53). A randomized, double‐blind clinical trial on 100 type 2 diabetic patients (55 were male and 45 were female) reported a reduction in HbA1c value from 8.6% at baseline to 7.42% after 12 weeks and thus indicated the potentiality of *Cichorium intybus* seed supplementation as an adjunct therapy in type 2 diabetes mellitus (54). A similar 4 weeks long randomized, double-blind, placebo-controlled study in 47 healthy subjects (8 were male and 39 were female; age range: 33-70 years) reported that the seed supplementation at 300 ml/day dose caused improvement in adiponectin level and fecal properties along with the antihyperglycemic activity, indicating that the supplement, which included inulin-type fructans, is effective in delaying diabetic mellitus onset and helps improving bowel movements (55).

## *Cinnamomum verum* J. Presl

*Cinnamomum verum* J. Presl (family: Lauraceae) is an evergreen plant with a height of 10-15 m which is local to Southern India and Srilanka. Apart from these places, it is widely available in other Asian, Australian, Caribbean and African countries but most notably in China, Indonesia, Madagascar, Vietnam and Burma. It is well-known as ‘Ceylon cinnamon’, ‘True cinnamon’, ‘Darchini’, ‘Dalchini’ and ‘Mexican cinnamon’. The name Ceylon came after the former name of Srilanka, its native place. Bark (after drying) is the most important part of the plant with remarkable medicinal values (56). Methanolic extract from *C. verum* can suppress the activity of α- glucosidase and α-amylase (57). According to the study, cinnamon aqueous extract also showed notable antidiabetic activity in alloxan induced diabetic rat models by reducing fasting blood sugar, triglycerides and total cholesterol when tested for thirty days long (57). Interestingly, a lower dose of cinnamon extract i.e. 200 mg/kg showed maximum antidiabetic efficacy. In another research, it was revealed that hydro-alcoholic extract of Cinnamon can ameliorate postprandial glycemia more than its aqueous extract (58). Cinnamon can also increase the uptake of glucose by upraising the number of insulin receptors, glucose transporter 4 and activating glycogen synthase to diminish glucose levels (56, 59). Cinnamon extract was also co-administered with other herbals to evaluate the synergistic activity on diabetic complications. Again, a combination of methanolic cinnamon extract along with green tea can also decrease blood glucose concentration and body weight significantly in streptozotocin induced diabetic rat models by showing synergism (56, 60). Among isolated phytochemicals, it is believed that cinnamon polyphenols like eugenol and pyrogallol can demonstrate antidiabetic properties by renovating beta cells which leads to hypoglycemic and hypolipidemic actions (56). According to Tulini et al. solid lipid microparticles (SLM) of proanthocyanidin rich cinnamon extract can improve the antidiabetic efficacy of foods (61). Again, cinnamaldehyde can ameliorate the uptake of glucose by upraising the amount of AKT2 and aortic nitric oxide synthase 3 (eNOS), insulin receptor substrate1 (IRS1) and p-85 regulatory subunit of PI3K (PI3K-P85) while concurrently abating the expression of NADPH oxidase 4 (NOX4) which eventually balance the increased glucose concentration (56, 59). Cinnamon supplementation at the dose of 500 mg showed prominent antidiabetic action in a 3 month long randomized, triple-blind placebo-controlled, parallel clinical trial in 138 type 2 diabetic patients (63 were male and 75 were female; age range: 30-80 years) by causing a reduction in all glycemic parameters, namely FPG by -13.1 ± 1.7 mg/dl, HbA1C by -0.27 ± 0.04%, 2HPP by -16.9 ± 2.5 mg/dl, insulin resistance (HOMA-IR) by -1.01 ± 0.11 and fasting insulin by -1.77 ± 0.41 mIU/L) with no side effects while a better glycemic control was observed in patients whose BMI values were greater than 27 (59). Another phase 1 clinical study on 30 healthy adults (50% were male; mean age: 38.8 ± 10.4 years; age range: range 21–58 years) showed that cinnamon had no significant toxicity or side effects (62). In a review of 8 randomized-controlled trials, it has been found that sole therapy of powdered or aqueous form of cinnamon, at different doses starting from 0.5 g to 5 g per day, ameliorated glycemic control in type 2 diabetic and prediabetic patients (impaired fasting glycemia or impaired glucose tolerance) (63).

## *Costus pictus* D. Don

*Costus pictus* D. Don (family: Zingiberaceae) is a rhizomatous medicinal herb which is popularly known as ‘Insulin plant’ for its strong antidiabetic efficacy. It demonstrated antidiabetic action by the inhibition of α-amylase and α–glucosidase activity (64). *C. pictus* can also improve the secretion of insulin in diabetic rat models along with improvement in glucose utilization (65). It has been found that upon the administration of aqueous extract of *C. pictus* to diabetic rats, *C. pictus* causes a marked reduction in blood glucose levels and an increase in plasma insulin level. Regulation of glucose homeostasis by improved peripheral glucose utilization, increased hepatic glycogen synthesis and/or decrease of glycogenolysis, inhibition of intestinal glucose absorption, and lowering of the glycaemic index of carbohydrates might be responsible for the antidiabetic effect of *C. pictus* (66). Earlier researchers also found that β-amyrin and methyl tetracosanate are the major bioactive phytoconstituents which exhibited ameliorated glucose uptake in 3T3-L1 adipocytes (67, 68). In another study conducted on *C. pictus*, β- L- Arabinopyranose methyl glycoside was reported responsible for antidiabetic property (69). Ingestion of the leaves of *C. pictus* by diabetic patients showed statistically significant reduction in their fasting and postprandial blood glucose levels, as per a cross-sectional clinical study (70).

## *Curcuma longa* L.

*Curcuma longa* L. (family: Zingiberaceae) also known as ‘Turmeric’ is a moderately tall perennial plant containing underground rhizomes. It is grown in tropical regions like Pakistan, China, Peru and India. The curcuminoids bisdemethoxycurcumin, curcumin and demethoxycurcumin were isolated from *C. longa* and were found to exhibit α-glucosidase inhibitory activity (71). Among the three curcuminoids, bisdemethoxycurcumin showed the most potent α-glucosidase inhibition (72). Besides, volatile oils extracted from both fresh and dried turmeric rhizomes showed potent glucosidase inhibitory activity in a dose-dependent mode, and dried rhizomes increased the glucosidase inhibitory action significantly. Potent α-glucosidase and α -amylase inhibitory activity was exhibited by Aromatic-Turmerone, the main volatile component in turmeric rhizome (73). Turmerin, a water-soluble protein found in turmeric rhizomes inhibits α-amylase and α-glucosidase activities. Thus, turmeric rhizomes exert inhibitory action against enzymes related to type 2 diabetes (71). A combination of *Curcuma longa* and *Allium sativum* at 2.4 g total dose showed prominent antihyperglycemic action in type 2 diabetic patients by reducing fasting blood glucose, 2 h postprandial glucose, HbA1C and body mass index levels without showing any side effects (74). In addition, in six clinical trials, treating type 2 diabetic patients with curcuminoids ranging from 0.25 g to 1 g per day also ameliorated glycemic control by decreasing fasting blood glucose, HbA1c, HOMA-IR (insulin resistance) levels and increasing adiponectin level without causing any major side effects. Improvement in diabetes-associated endothelial dysfunction and hyperlipidemia was observed as well (75).

## *Cryptolepis sanguinolenta* (Lindl.) Schltr.

*Cryptolepis sanguinolenta*, (Lindl.) Schltr. (family: Apocynaceae) is a scrambling thin-stemmed shrub indigenous to West Africa which is commonly found in tropical rainforests, thickets, and mountainous ecologies (76). It was found to reduce the intestinal absorption of glucose and its transport from the gut significantly in a dose-dependent mode in the normoglycemic rats (76, 77). The study also revealed that treatment with *C. sanguinolenta* increased the size of β cells which might have improved the production and activity of insulin resulting in reduced blood glucose levels. Additionally, *C. sanguinolenta* also increases the uptake of glucose by 3T3-L1 cells, and improves insulin-mediated disposal of glucose. The hypoglycemic activity exhibited by the extract of *C. sanguinolenta* may be due to the presence of its alkaloid constituents. It has been reported that insulin resistance is reduced by alkaloids in mice and high fat-fed rats. They can activate AMP-activated protein kinase in 3T3-L1 adipocytes and L6 myotubes and promote the translocation of GLUT4 in L6 myotubes in a manner that is independent of phosphatidylinositol 3-kinase. Besides, they also improve the uptake of glucose in HepG2 and 3T3-L1 cells. In addition, they probably suppress the activity of the α-glucosidase enzyme and cause a reduction in the absorption of glucose. It has also been reported to significantly reduce the plasma levels of IL-6 with increased insulin sensitivity. This mechanism of action might also be responsible for the hypoglycemic activity of alkaloids-containing *C. sanguinolenta* stem extract (77). Cryptolepine, an indoloquinoline alkaloid purified from *C. sanguinolenta*, was found to reduce plasma glucose level significantly in a mouse model of diabetes, and in that model, it was approximately as effective as ciglitazone. It was suggested that cryptolepine works directly at the cellular level to enhance the glucose transport in 3T3-L1 cells and thus causes a reduction in blood glucose level (78).

## *Euclea undulate* Thunb. var. myrtina

*Euclea natalensis* Thunb. var. myrtina (family: Ebenaceae) is a multi-stemmed, dioecious shrub or little tree growing up to about 6 m height. It is distributed in Botswana, Zimbabwe, Namibia, Swaziland, Mozambique and South Africa. Its crude acetone root bark extract was found to show antidiabetic activity in type 2 induced diabetic rat models (79). Besides, the plant extract of *E. undulata* was found to inhibit α-glucosidase and α-amylase activity (80). Past studies have revealed the presence of epicatechin and α-amyrin-3O-β-(5-hydroxy) ferulic acid in the crude acetone extract of the root bark of *E. undulata*. It has been reported that epicatechin may have the ability to lower blood glucose levels and α-amyrin-3O-β-(5-hydroxy) ferulic acid can inhibit α –glucosidase (81).

## *Gymnema sylvestre* R. Br.

*Gymnema sylvestre* R. Br. (family: Asclepiadaceae) is an evergreen, woody climber and endogenous plant which is widely available in central and southern India and in the southern part of China, Sri Lanka, Malaysia and tropical Africa, Malaysia (82). *G. sylvestre* can improve average blood glucose levels in animal models and can stimulate insulin secretion from the MIN-6, HIT-T15 and RINm5F β-cells by upraising membrane permeability (83). *Gymnema sylvestre* is thought to act by several mechanisms including regeneration of islet cells, increase in the secretion of insulin and glucose utilization by insulin-dependent pathway, increase the phosphorylase enzyme activity, decrease in gluconeogenic enzymes and sorbitol dehydrogenase, reduction of glucose absorption from the gut wall (84). Antihyperglycemic compounds like gymnemagenin and gymnemic acids were discovered by LC/MS analysis from the ethanol extract of *G. sylvestre* leaves which demonstrated blood glucose level lowering activity in rat models (83). Gymnemic acid is a complex mixture of several (more than seventeen) saponins which are mainly dammarene and oleanane. 3-O-β-D-glucopyranosyl (1-6)-β-D-glucopyranosyloleanolic acid 28-O- β-Dglucopyranosyl ester, longispinogenin 3-O-β- D-glucuronopyranoside, oleanolic acid 3-O-β-D-xylopyranosyl(1-6)-β- D-glucopyranosyl(1-6)-β-D-glucopyranoside and 3-O-β-D-glucopyranosyl (1-6)-β-D-glucopyranosyl oleanolic acid 28-β -D-glucopyranosyl (1-6)-β-Dglucopyranosyl ester and 21 β-benzoylsitakisogenin 3-O-β- D-glucuronopyranoside 3-O-β-D-xylopyranosyl (1-6)-β-D-glucopyranosyl (1-6)-β-D-glucopyranosyl oleanolic acid 28-O-β -D-glucopyranosyl ester are contributing to major oleanane-triterpene glycosides. There are also seven novel dammarane saponins from the leaf extract of *G. sylvestre* known as gymnemasides I-VII. The introduction of gymnemic acid IV can decrease whose efficacy is comparable to the commercially available drug glibenclamide. A recent study also revealed that crystallographic investigation of gymnemagenin certainely indicated its good gelling with the target protein’s crystallographic constitution (aldose reductase, dipeptidyl peptidases, fructose 1,6-bisphosphate, glucokinase, 11β-hydroxysteroid dehydrogenase, cytochrome 450, protein kinase B, tyrosine phosphatases, Insulin receptor substrate, cholesteryl ester transfer protein, glutamine fructose- 6-phosphate amidotransferase, AMP-activated protein kinase and Glucose transporter) which contribute to its carbohydrate management property (83). In addition, the leaf extract of *Gymnema sylvestre* at 400 mg b.i.d dose lessened fasting blood glucose levels by 11%, postprandial blood glucose levels by 13% and HbA1c value by 0.6% in a 3 month long open-label trial consisting of 65 type 2 diabetic patients (85). Such lessening of the fasting blood glucose levels and post-prandial blood glucose levels were additionally consolidated by two other open-label trials on diabetic patients where the leaf extract of *G. sylvestre* were administered in the dose of 6–10 g for 15–21 days (86, 87). Moreover, in a 18-20 month-long controlled, open-label study on 22 subjects suffering from type 2 diabetes, 400 mg/day supplementation of *G. sylvestre* leaf extract exhibited a better reduction in blood glucose, glycosylated haemoglobin and glycosylated plasma protein levels compared to conventional therapy of tolbutamide or glibenclamide alone. Interestingly, of the total 22 patients participating in the trial, 5 patients could maintain blood glucose homeostasis with 400 mg/day dose of *G. sylvestre* alone even after ceasing their conventional drug therapy (88). A similar open-label study on 27 type 1 diabetic patients observed that the leaf extract supplementation reduced the levels of glycosylated plasma protein and serum amylase and increased serum C-peptide levels in contrast to the conventional therapy alone through the possible mechanism of regenerating the residual beta cells of the pancreas (89).

## *Gynura divaricate* (L.) DC.

*Gynura divaricate* (L.) DC. (family: Asteraceae) is a traditional Chinese herbal plant locally known as ‘Bai Bei San Qi’. It also cultivates in the eastern and northern Taiwan coasts though is widely found in various part of Asia (90). *G. divaricate* can improve glucose metabolism in animal models including mice and rats. A study also revealed its low toxicity profile in both *in vivo* and *in vivo* testing along with significant reduction of fasting serum glucose and improved pancreatic damage (91). Among hypoglycemic phytoconstituents of the plant aerial part, a few major compounds are nystose, β-D-fructofuranose, 1-kestose, sucrose and 1F-β-fructofuranosylnystose which are fructooligosaccharides. The hexose transport assay showed that Nystose delivered the most powerful hypoglycemic activity among these five isolated phytocompounds (92). PKM1/2, PI3K, p-AKT, and GLUT4 play a vital role in the insulin signaling pathway in diabetes. High blood glucose-induced cell death and senescence in nucleus pulposus cells are inhibited by the activation of the PI3K/AKT signaling pathway. PKM1/2 is involved in glycolysis, and GLUT4 is a glucose transporter which is present on the cell membrane. Past studies also suggested that increasing GLUT4 expression promotes the glucose uptake and utilization. It has been found that *Gynura divaricate* increases the expression levels of PKM1/2, PI3Kp85, p-AKT, and GLUT4 resulting in the reduction of blood glucose levels (93).

## *Hordeum vulgare* L.

*Hordeum vulgare* L. (family: Poaceae) is an annual herbaceous monocotyledonous grass commonly known as ‘Barley’ which originated in the Fertile Crescent including Israel, Jordan, Syria and southern Turkey to Zagros Mountains in Iran (94, 95). It contains high levels of dietary fiber such as β-glucans whose oral administration into type 2 diabetic and high‐fat diet induced obese mice resulted in a significant lowering of blood glucose level (96). The underlying mechanism is thought to be the suppression of sodium‐glucose transporter‐1 expression in the intestinal mucosa. Besides, it also promotes glycogen synthesis and inhibits fat accumulation in the liver, and depresses macrophage infiltration and the production of pro‐inflammatory cytokines (97). It was also found to promote glucose uptake and reduce gluconeogenesis by downregulating some genes responsible for gluconeogenesis. Barley is also rich in magnesium acting as a co-factor for more than 300 enzymes as well as for those which are involved in glucose metabolism and insulin secretion. It has been observed that regular consumption of whole grains can lower the risk of type II diabetes by 31%. This could be due to its high fiber content (98). β-glucans from barley also improved glycemic control in diabetic patients, mainly by abating postprandial blood glucose levels, according to multiple clinical studies. It has been suggested through clinical trials that high β-glucans containing foods have lower glycemic index (GI) and diabetic people should substitute the high-GI foods in their diet with low-GI foods in order to control the disease (99, 100).

## *Larrea tridentata* (Sessé & Moc. ex DC.) Coville

*Larrea tridentata* (Sessé & Moc. ex DC.) (family: Zygophyllaceae), known as Coville or ‘Creosote bush’ is a highly brunched and evergreen shrub from Zygophyllaceae which is widely available in North American warm deserts and Mexico (101). It contains Masoprocol, a lipooxygenase inhibitor as the major antihyperglycemic compound which decreased plasma glucose level in type-2 diabetic mice models without changing the concentration of plasma insulin. Additionally, it has been shown to improve oral glucose tolerance and enhance insulin activity in lowering the plasma glucose levels in masoprocol-treated db/db mice (102).

## *Lobelia chinensis* Lour.

*Lobelia chinensis* Lour. (family: Campanulaceae), known as ‘Asian Lobelia’ or ‘Chinese Lobelia’ is distributed throughout China, Taiwan, Korea, and Japan. It is a plant from which two new pyrrolidine-type alkaloids, radicamines A and radicamines B, were found to inhibit α-glucosidase activity and demonstrate antidiabetic efficacy (103). Besides, it has also been reported that the active ingredients of 5-hydroxymethylfural and acacetin in *L. chinensis* has been shown to promote the secretion of insulin, improve insulin resistance, and stimulate the utilization of glucose by acting on GSK3B, MAPK, INR, and dipeptidyl peptidase-4 (DPP4) (104).

## *Lupinus perennis* L.

*Lupinus perennis* L. (family: Fabaceae) is a perennial herb which is abundantly found in Canada and USA (105). It is a plant from the leaves of which the compounds lupanine, 13-a-OH lupanine, and 17-oxo-lupanine were extracted and enhanced the secretion of insulin from isolated rat islets in a glucose-dependent manner. It was assumed that these quinolizidine alkaloids can increase insulin release by reducing K^+^ permeability in the β-cell plasma membrane. The fact that 13-a-OH lupanine and 17-oxo-lupanine stimulate insulin secretion only at high glucose concentrations indicates that it would reduce the risk of hypoglycemia which could be of additional value when considering their potential use in the treatment of type 2 diabetes (106).

## *Matricaria chamomilla* L.

*Matricaria chamomilla L.* (family: Asteraceae), commonly known as ‘German chamomile’, ‘Hungarian chamomile’ or ‘wild chamomile’, is a herbaceous plant natively distributed in European and West Asian regions (107). Its aerial part’s ethanol extract dose-dependently lessened postprandial blood glucose levels and showed protective action on pancreatic β-cells of streptozotocin (STZ)-induced diabetic rats. The same study also reported a significant reduction in oxidative stress related to hyperglycemia (108). A similar study on STZ-induced diabetic rats showed that a 21 day long 200 mg kg-^1^ body weight dose of *M. chamomilla* leaf extract significantly diminished the fasting blood glucose levels by 62.2% (109). HbA1C and blood glucose values were also reduced in STZ-induced female fertile diabetic rats after administering the plant’s aerial part-derived ethanolic extract (110). In addition, another animal study reported the effectiveness of *M. chamomilla* flower extract and the isolated compounds quercetin, esculetin, umbelliferone and luteolin in preventing the progression of hyperglycemia. It was observed that quercetin and esculetin moderately inhibited the enzymatic activity of sucrase in rats and all the compounds halted sorbitol from accumulating in the erythrocytes of humans. Quercetin and the hot water extract also suppressed blood glucose levels in a 21 days long feed test on STZ-induced diabetic rats. Furthermore, esculetin diminished hyperglycemia in a disaccharide loading test on mice. It was also reported that the extract exhibited good inhibitory activity against the aldose reductase enzyme (111). Apigenin, which is another compound isolated from the plant, showed antihyperglycemic action by causing increment in blood insulin and diminution in blood glucose levels in alloxan induced diabetic mice (112). In an 8 week long randomized controlled trial on 64 type 2 diabetic patients (12 were male and 52 were female; age range: 30-60 years), chamomile tea in the dose of 3 g/150 mL thrice a day showed a significant diminution in the HbA1C level by 5.01%, HOMA-IR level by 39.76%, and serum insulin level by 32.59% compared to baseline values and caused improvement in antioxidant activity (113). Another 4 week long randomized-controlled trial on 50 type 2 diabetic patients observed that twice-daily infusion of 10g/100 ml chamomile as a supplementation significantly improved glycemic control by lessening fasting blood glucose and 2h postprandial blood glucose levels. Moreover, lipid profile was also ameliorated in the patients (114).

## *Momordica charantia* L.

*Momordica charantia* L. (family: Cucurbitaceae) is a flowering vine cultivated in Asia including Bangladesh, India and in other regions like East Africa and South America as well. The fruit has a distinct bitter taste for which this plant is known as bitter gourd which is also well known as ‘Korolla’, ‘Karela’, ‘Bitter melon’ or ‘Balsam pear’. The bitterness becomes more intensified when it ripens. The plant produced notable antidiabetic and hypoglycemic actions which ascertain the adjuvant use of the plant along with conventional commercialized drugs (115). The oral consumption of the juice of *M. charantia* seeds showed prominent hypoglycemic activity in streptozotocin induced type 1 diabetic rat models. There are many bioactive phytocompounds isolated from *M. charantia* producing remarkable antidiabetic activity. Among saponins, 3-hydroxycucurbita-5, 24-dien-19-al-7, 23- di-O-β-glucopyranoside and Momordicine- II were extracted from corolla exhibiting promising insulin-releasing properties in MIN6 β-cells. Charantin, a cucurbitane type triterpenoid extracted from the same plant has also showed tremendous antidiabetic activity which is even more potent than standard oral hypoglycemic drug tolbutamide (115). Polypeptide-p or p-insulin, insulin-like hypoglycemic protein type substance which demonstrated blood glucose-lowering activity in human upon subcutaneous administration was isolated from corolla. The insulin mimicking activity of Polypeptide-p can be considered as a plant based alternative of insulin in type 1 diabetic patients (115). Vicine, a glycol alkaloid is another isolated compound from *M. charantia* which can promote hypoglycemia in non-diabetic fasting rat models upon intraperitoneal administration (115). Among other isolated compounds, Momordicoside U showed moderate activity during *in vitro* insulin secretion property screening and 5β,19-epoxy-3 β,25-dihydroxycucurbita-6,23(E)-diene and 3 β,7 β,25-trihydroxycucurbita-5,23(E)-dien-19-al both revealed hypoglycemic activity in diabetes induced male mice models (116, 117). A meta-analysis study of ten clinical trials conducted in 1045 type 2 diabetic patients observed that *M. charantia* possessed significant glycemic control improving ability since it lessened FPG, HBA_1c_ and PPG levels without causing any side effects. In addition, prediabetic subjects also had a reduction in their FPG levels (118, 119). Such action is reported to occur because of increased insulin secretion, as per another randomized-controlled trial. Moreover, anthropometric parameters, namely weight, body mass index, waist circumference, fat percentage were also decreased (120). Another trial on maturity-onset diabetic patients reported the antihyperglycemic action of *M. charantia* since it decreased mean blood glucose levels in fasting conditions as well as at 1, 2 and 12 hours after oral intake of 50 g glucose (121).

## *Moringa oleifera* Lam.

*Moringa oleifera* Lam. (family: Moringaceae) is a perennial angiosperm plant native to Asia and greatly found in Malaysia and other tropical countries. Local name of *M. oleifera* is ‘Sajna’, ‘Soanjna’ and ‘Sohanjna’ and the english name is ‘Drum stick tree’ (122). It is a plant whose leaf’s alcoholic extract along with its antidiabetic phytocompounds like flavonoids, alkaloids, tannins, steroids and glycosides is assumed to be effective to treat diabetic complications. Quercetin and kaempferol, two major phytoconstituents, isolated from *M. oleifera* notably reduced serum glucose (33.34%) along with augmentation in serum insulin level when introduced to diabetic rat models for four weeks (123). In another study, moringinine, quercetin and chlorogenic acid, notable phytochemicals extracted from this plant were introduced to diabetic rat models to evaluate antidiabetic efficacy. The outcome showed alleviated serum glucose, total cholesterol and triacylglycerol level at a dose of 150 mg/kg after 21 days of care. In addition, in diabetic rats, it also restored the normal histological structure of the pancreas (124). Past studies have indicated that the glucose uptake in the rat soleus muscle is stimulated by kaempferol *via* the PI3K and PKC mechanisms. When administered orally, kaempferol was found to reduce fasting blood glucose levels significantly and serum HbA1c levels besides improving insulin resistance. Additionally, Quercetin blocks the transport of fructose and glucose by GLUT2 in the brain and promotes the translocation and expression of GLUT4 in skeletal muscle (125). In addition, in a randomized control design trial, promising inhibition in the increment of serum glucose after 2h of 75g oral glucose intake was observed after taking capsules processed with *M. oleifera leaf* in a population pool of 18-55 years old (126). The efficacy of *M. oleifera* in twenty diabetic and ten healthy individuals of 35 to 60 years old was evaluated in another clinical trial. The concentration of glucose, triglycerides, glycosylated hemoglobin, total cholesterol and low-density lipoprotein cholesterol decreased significantly while high-density lipoprotein cholesterol was upraised. This hypoglycemic efficacy has been assumed to be attributed to phenols, tannins, flavonoids, alkaloids and carotenoids (122). In another study, an assessment was conducted to evaluate the ability of *M. oleifera* leaf powder to inhibit the activity of α-amylase *in vitro*. The study found that M. oleifera leaf powder decreased α-amylase enzyme activity by 68.2 ± 3.2% (127). The study also further evaluated *in vivo* activity of the leaf powder on postprandial blood glucose levels in the subjects of Saharawi refugee camps (17 diabetic and 10 healthy individuals). The study displayed that administration of 20g leaf powder improved postprandial glycemic index at 90, 120 and 150 min. as well as improved the mean glycemic index in diabetic patients compared to the control group which indicates the candidacy of *M. oleifera* as an antihyperglycemic herbal drug (127).

## *Morus alba* L.

*Morus alba* L. (family: Moraceae), known as ‘Mulberry’ is a quickly growing tree growing as long as 20 m. It is native to China though cultivated sporadically in Japan and Korea (128). Three major compounds, namely Moracin M, steppogenin-4′-O-β-D-glucosiade and mullberroside A showing efficacy in alloxan induced mice models by demonstrating hypoglycemic efficacy and decreasing fasting blood glucose level were isolated from the plant (129). Moreover, the alkaloids extracted from the leaves of mulberry were found to exhibit hypoglycemic effects in streptozotocin- (STZ-) induced diabetic mice. It has been reported that 1-deoxynojirimycin (DNJ), a mulberry alkaloid, reduces the activity of α-glucosidase by competitive inhibition. Upon oral administration of starch and sucrose in Kunming mice, flavonoids from mulberry leaf reduced blood glucose level and inhibited α-glucosidase activity. In the laboratory experiment, two flavonoids (isoquercitrin and astragalin) were found to inhibit α-glucosidase activity. Polysaccharides isolated from the leaves of mulberry were reported to reduce plasma glucose level, improve glucose tolerance, increase the hepatic glycogen content, and inhibit α-glucosidase activity. The extracted polysaccharides α-arabinose, α-xylose, α-glucose, α-rhamnose, and α-mannose were found to repair pancreatic β-cells, resulting in increased insulin secretion and reduced accumulation of liver fat in diabetic rats (130). A randomized, placebo-controlled study on 10 type 2 diabetic (age range: 59 –75 years) and 10 healthy subjects (age range: 24 – 61 years) involving the ingestion of 1 g leaf extract of *Morus alba* showed remarkably reduced blood glucose levels after 2 hours in comparison to the placebo group (131). Another trial showed the effectiveness of the leaf extract in suppressing insulin and postprandial blood glucose levels (132). In addition, fasting blood glucose was better reduced by *M. alba* compared to glibenclamide in 24 type 2 diabetic patients (24 male; age range: 40-60 years), as per a 30 day long randomized controlled trial (133). Moreover, a 25% inhibition in carbohydrate absorption of healthy subjects was observed in another crossover trial, suggesting that *M. alba* could also be used as a supplementation in the treatment of type 2 diabetes (134). 1-Deoxynojirimycin (DNJ) from the leaves of *M. alba* in 0.8 and 1.2 g doses also notably reduced the postprandial blood glucose levels and insulin secretion of 24 healthy subjects (mean age: 25.3 ± 0.7 years) in a randomized controlled trial. Such efficacy advocates for the use of 1-Deoxynojirimycin as a dietary supplement in the treatment of diabetes mellitus (135).

## *Nelumbo nucifera* Gaertn.

*Nelumbo nucifera* Gaertn. (family: Nelumbonaceae), commonly known as ‘Chinese water lily’, ‘Indian lotus’ and ‘Sacred lotus’, is a large aquatic rhizomatous perennial plant (136). Nuciferine, an alkaloid was extracted from the plant through identifying by NMR spectroscopy and was found to increase insulin secretion in both isolated islets and INS-1E cells. It was found that nuciferine stimulates both the first phase and the second phase of insulin secretion. These results indicated that the nuciferine acts by closing K-ATP channels and also through stimulation of K-ATP channel-independent amplification pathways. Besides, it shows less cytotoxicity than Glibenclamide (137). In addition, *N. nucifera* seeds achieved a low glycemic index (GI) in a randomized crossover trial on healthy subjects (138).

## *Nigella sativa* L.

*Nigella sativa* L. (family: Ranunculaceae) is a herbaceous plant which occurs in several southern Mediterranean and Middle Eastern countries. The seeds of *Nigella sativa* are known by the name ‘Black seed’ or ‘Black cumin’. Its seed’s ethanolic extract was found to enhance insulin secretion, stimulate proliferation of pancreatic β-cells, and enhance glucose uptake in muscle and fat cells (139). It has been suggested that *Nigella sativa* exhibits its hypoglycemic effect due to the presence of thymoquinone, dithymoquinone, linoleic acid and oleic acid which might be responsible for stimulating pancreatic β-cells causing insulin secretion, reducing hepatic gluconeogenesis, and inducing insulin sensitivity in peripheral tissue. A placebo-controlled participant blinded clinical study on 114 type 2 diabetic patients found that *N. sativa* supplementation plays an important role in the amelioration of oxidative stress, the latter being responsible for diabetes mellitus pathogenesis. It has been found that the former does so by improving total antioxidant capacity, glutathione and superoxide dismutase values. The same study perceived that reduction in insulin resistance was also significant in the diabetic patients in the group taking *N. sativa* supplementation compared to the placebo group (140). Supplementation of *Nigella sativa* in type 2 diabetes patients was found to improve fasting blood glucose, HbA1c, total- cholesterol, and LDL level significantly (33). As such, a systematic review done to assess the effect of *N. sativa* on type 2 diabetes mellitus has also suggested that it could be adjunctively used with other oral antidiabetic medications to manage the disease (141).

## *Panax ginseng* C. A. Meyer

*Panax ginseng* C. A. Meyer (family: Araliaceae) is also known as ‘Asian ginseng’. It is a plant that has been reported to modify blood glucose levels by increasing insulin sensitivity, ameliorating the function of pancreatic β-cells, and stimulating glucose uptake by elevating the production of glucose transporters (GLUT). The berry extract of *P. ginseng* stimulates the β-cell proliferation leading to increased insulin secretion to control the level of blood glucose in streptozotocin (STZ) -induced diabetic mice. Besides, it also results in improved sensitivity to insulin in C57BL/6 mice over 15 months old (142). Ginseng is known to contain ginsenosides, a group of steroidal saponins, including neutral ginsenosides and malonyl ginsenosides. Ginseng and neutral ginsenosides were found to lower blood glucose, increase insulin sensitivity, regulate lipid metabolism, and reduce body weight (143, 144). Administration of malonyl ginsenosides in high fat diet/streptozotocin diabetic rats was found to remarkably reduce fasting blood glucose levels, improve glucose tolerance and insulin sensitivity without affecting body weight (143, 144). The study results suggest that malonyl ginsenosides could be used to treat type-2 diabetes. In addition, a meta-analysis of sixteen clinical trials revealed that Ginseng caused a significant reduction in fasting blood glucose levels in both diabetic and non-diabetic patients, while the other glycemic parameters were contradictory in terms of outcomes (145). Besides, a randomized, placebo‐controlled, double-blind study in 20 type 2 diabetic patients (mean age: 51.5 ± 1.9 years) observed about 45% reduction in HOMA-IR in the group which was given *P. ginseng* supplementation compared to 12% reduction for the placebo group (146). Moreover, combinatory administration of *Panax ginseng* and *Panax quinquefolius* in a 12 week long randomized controlled trial on 80 type 2 diabetic subjects (49 were male and 31 were female) reduced another glycemic control parameter, namely HbA1c by −0.35 ± 0.1% along with blood lipid parameters (total cholesterol, LDL-C, triglycerides) and 24-hour systolic blood pressure without causing any side effects (147).

## *Pandanus amaryllifolius* Roxb.

*Pandanus amaryllifolius Roxb.* (family: Pandanaceae) is a shrub native to Thailand. This plant is also known as *Pandanus odorus*. Local name of the plant is ‘Toei-hom’ (148). In recent studies, *P. amaryllifolius* exhibited very prominent hypoglycemic activities. The root extract of the plant evidently lowered blood glucose levels in streptozotocin induced mice models. The leaf extract could also show promising antihyperglycemic activity by stimulating insulin production and glucose uptake along with inhibition of α-glucosidase enzyme (148). 4-Hydroxybenzoic acid is the major hypoglycemic phytoconstituents isolated from the aqueous extract of this plant root. Upon oral administration, this moiety upraised serum insulin and liver glycogen level in normal rat models (149). A clinical study performed on 30 healthy subjects (15 were male and 15 were female; age range: 15-25 years) showed that intake of 30 g of *P. amaryllifolius* containing tea caused a significant lessening of postprandial blood glucose (148).

## *Punica granatum* L.

*Punica granatum* L. (family: Punicaceae) locally known as ‘Dalim plant’ is a plant native to some Asian countries including India, Bangladesh, Iran and Malaysia along with some other countries of America and European continents, such as United States of America, Peru, Turkey, etc. The fruit of the plant (commonly known as ‘Dalim’ in Asian countries) is popularly consumed as fresh form along with processed juice, jam, paste and wine (150). The fruit aqueous extract of *P. granatum* can notably decrease fasting glucose level along with promising increment in the expression levels of Glut-4, Glut-2, Akt and IRS-1 followed by improved glucose uptake and its storage in alloxan induced male Wistar rat models (151). It is blessed with several polyphenolic compounds like punicalagin, valoneic acid dilactone, anthocyanin, phenolic and non-phenolic Acids, Glutenins and Tannins (151). Among those, valoneic acid dilactone is the main antidiabetic principle which showed its antidiabetic efficacy by inhibiting the activity of aldose reductase enzyme in a dose dependent pattern. Protein tyrosine phosphatase 1B (PTP1B) was also inhibited by valoneic acid dilactone which can also ameliorate the level of blood glucose in alloxan induced diabetic rat models. Other possible mechanisms attributed to antidiabetic action of valoneic acid dilactone may be attributed to improved insulin secretion from rom pancreatic β cells or its release from the bound form along with insulin-mimetic actions or amended glucose utilization technique (150). In addition, an 8-week long double blind randomized-controlled clinical study on 52 type 2 diabetic patients with obesity (26 were male and 26 were female; age range: 30-50 years) found that supplementation of *P. granatum* significantly reduced fasting blood glucose from 161.46 mg/dl to 143.50 mg/dl. An increase in GLUT-4 gene expression was also observed in the patients (152). Furthermore, another single-blind, randomized-controlled clinical study conducted on 44 type 2 diabetic patients (23 were male and 21 were female; age range 56 ± 6.8 years) found that the juice of *P. granatum* significantly ameliorated oxidative stress, suggesting that it’s consumption could retard the onset of oxidative stress associated diabetes mellitus (153).

## *Pongamia pinnata* (L.) Pierre

*Pongamia pinnata* (L.) Pierre (family: Fabaceae) is a medium-sized glabrous tree, commonly known as ‘Karanja’ in Hindi, ‘Indian Beech’ in English, and ‘Pongam’ in Tamil. It occurs throughout India, and mainly found in tidal forests of India. The flowers of *P. pinnata* reportedly possessed anti-hyperglycemic and anti-lipidperoxidative properties (154). It has been found that oral administration of the aqueous (PPAE) and ethanolic (PPEE) extracts of the leaves of *P. pinnata* PPEE in alloxan diabetic rats resulted in a pronounced reduction in the plasma glucose level. This may be attributed to the enhancement of the effect of insulin by increasing the insulin release from the pancreatic β-cells or its release from the bound insulin. The significant glucose-lowering effect of PPAE and PPEE could also result from increased peripheral glucose utilization (155). Karanjin, one of the isolated compounds from this plant was reported to possess hypoglycemic activity in normal and in alloxan-induced diabetic rats. Pongamol and karanjin extracted from the chloroform-soluble fraction of the ethanolic extract of *P. pinnata* fruits exhibited significant glucose-lowering activity (156). The underlying mechanism of anti-hyperglycemic activity of the compounds may be attributed by the inhibition of PTPase-1B, a major mediator of insulin signaling and insulin resistance (156).

## *Psacalium peltatum* (Kunth) Cass.

*Psacalium peltatum* (Kunth) Cass. (family: Asteraceae) is a plant natively grown and known as ‘Matarique’ in Mexico. Peltalosa, an ulopyranose compound, has been obtained from the roots and rhizomes of *P.peltatum* which has showed anti-hyperglycemic activity on mice with mild diabetes, although the efficacy decreased on mice models with severe diabetes (157). The hypoglycemic effect of the compound was reported to be similar to tolbutamide, and the possible underlying mechanism of action could be attributed to enhanced secretion of insulin from the islets of Langerhans or an increased utilization of glucose by peripheral tissues (157).

## *Silybum marianum* (L.) Gaertn

*Silybum marianum* (L.) Gaertn (family: Asteraceae), also known by the common name ‘Milk thistle’ is an annual or biennial herb native to the Mediterranean regions of Europe, North Africa and the Middle East and in some parts of USA (158, 159). Its major component is silymarin, a mixture of silibinin (silybins A and B), isosilybin (isosilybins A and B), silychristin and silydianin (160). Administration of silymarin in patients with type 2 diabetes resulted in a significant reduction in HbA1c level, fasting plasma glucose (FPG), daily blood glucose average and glucosuria, daily insulin requirement, fasting insulin, as well as an increase in serum glutamic oxaloacetic transaminase (SGOT), serum glutamic pyruvic transaminase (SGPT) and HDL levels. When silymarin was supplemented with glibenclamide in type 2 diabetes patients, a reduction in postprandial hyperglycemia was also observed (33). A flavonolignans, silychristin A, extracted from *Silybum marianum* demonstrated a marked reduction of both postprandial and/or fasting hyperglycemia and improvement of the function of β-cells in STZ-induced T1DM. It has been suggested that silychristin A exerts its glucose-lowering effect by protecting the β-cells from oxidative stress-induced damage and blocking the activity of the α-glucosidase enzyme (159).

## *Swertia chirayita* Buch Ham.

*Swertia chirayita* Buch Ham., (family: Gentianaceae) locally named as ‘Chirayata’, ‘Chirayta’ and ‘Chiretta’ is a popular medicinal herb native to temperate Himalayan region (161). Promising antidiabetic efficacy of *S. chirayita* with improved insulin secretion was reported during cell line based evaluation technique using insulin secretion from monolayers of BRIN-BD11 clonal pancreatic cells (162). There are several antidiabetic compounds found in this species exerting prominent antidiabetic efficacy. According to 163, 1,5,8-trihydroxy-3- methoxyxanthone extracted from the aerial parts and roots of the *S. chirata* can demonstrate antidiabetic efficacy by lowering blood sugar levels (163). Gentianine, another antidiabetic compound of this plant is the active metabolite of swertiamarin and is believed to attribute to the efficacy of swertiamarin (164). Promising amelioration in adipogenesis associated expression of PPAR-γ, GLUT-4 and adiponectin by gentianine administration expressed that the compound is responsible for antidiabetic efficacy of swertiamarin. Magniferin, a potent phytoconstituent found in *S. chirata* can also exhibit antihyperglycemic potentiality by exhibiting glucosidase and 2,2-diphenyl-1-picrylhydrazyl radical inhibition action. Besides, as a co-therapy with metformin and gliclazide it cured renal injury symptoms due to diabetic neuropathy (165). Moreover, compounds, found in *S. chirata* like amarogentin is used in the preparation of different forms of commercially available drugs to treat diabetic complications (166). A 30 day long clinical study done on 12 type 2 diabetic patients found that ingestion of *S. chirayita* in grounded powder form caused 14.5% reduction in blood glucose level. A reduction was also noticed in lipid profile (total cholesterol by 8.6%, LDL-c by 14.4% and, tryglycerides by 10.5%) (167).

## *Syzygium cumini* (L.) Skeels

*Syzygium cumini* (L.) Skeels (family: Myrtaceae) is known as ‘Jamun’, ‘Jambul’ and ‘Jambol’ in India and Malaya. The seeds of *S. cumini* are thought to lower blood sugar levels by increasing either insulin secretion from β-cells of the islets of Langerhans of Pancreas or its release from the bound form. Mycaminose, isolated from the seeds of *S. cumini* was found to produce a remarkable reduction in blood glucose level (168). It was suggested that the mode of action of mycaminose is similar to Glibenclamide, a commercially available anti-diabetic drug (168). In a double-blind randomized controlled trial involving 99 type 2 diabetic patients, 10 g daily *Syzygium cumini* supplementation significantly lessened fasting blood glucose by 30%, post-prandial blood glucose by 22% and HbA1c value from 8.99 ± 1.39% to 8.31 ± 1.40% after 90 days (169).

## *Tinospora cordifolia* (Willd.) Miers

*Tinospora cordifolia* (Willd.) Miers (family: Menispermaceae) is commonly known as ‘Guduchi’ or ‘Amrita’ and is found in the Indian subcontinent and China. It has exhibited blood and urinary glucose-lowering activity along with suppression in the increase of blood glucose level in animal models (170, 171). It is considered as an antidiabetic herbal drug in the Indian Ayurvedic Pharmacopoeia too due to its alkaloids, diterpenoids and glycosidic constituents. Among the alkaloids, magnoflorine was found to be the most potent α-glucosidase inhibitor (172). Besides, a norclerodane diterpenonoid, tinosporaside, extracted from *T. cordifolia* possessed 28% antihyperglycemic activity when it was compared with Metformin 20.6% in diabetic rat models (173). It has been found that the isoquinoline alkaloid rich fraction (AFTC) isolated from the stem of *T. cordifolia* (AFTC) significantly reduced the synthesis of glucose in rat hepatocytes like insulin did and it also stimulated secretion of insulin in RINm5F cells like tolbutamide. The underlying mechanism may be attributed to the promotion of insulin release and insulin-mimicking activity (174). In addition, the powdered stem of *T. cordifoia* at the oral dose of 50 mg/kg of body weight significantly reduced the fasting blood glucose and HbA1c levels by 9% and 14%, respectively in type 2 diabetic patients (175).

## *Trigonella foenum-graecum* L.

*Trigonella foenum-graecum* L. (family: Fabaceae) known as Fenugreek or Methi is a legume and a popular seasoning worldwide to improve the taste and flavor of food (176). Its seed water-soluble compound GII extract when administered for 15 days in the subdiabetic and moderately diabetic rabbits and for 30 days in the severely diabetic rabbits resulted in the elevation of hepatic and muscle glycogen content, stimulated hexokinase, glucokinase, pyruvate kinase, malic enzyme, glucose-6-phosphate dehydrogenase, superoxide dismutase, glutathione peroxidase, and reduced the activity of glucose-6-phosphatase, sorbitol dehydrogenase, aldose reductase. Partially damaged pancreatic cells were also regenerated following the administration of GII (177). Trigonelline, nicotinic acid and coumarin are antidiabetic phytochemicals that were isolated from Fenugreek seed. These three antidiabetic compounds extracted from fenugreek showed prominent efficacy in alloxan induced severe and moderate diabetic rabbit models (178). A meta-analysis on 10 clinical trials conducted on type 2 diabetic patients revealed that fenugreek can significantly improve glycemic control by altering parameters, namely fasting blood glucose level by -0.96 mmol/l, HbA1c value by -0.85% and 2 hour postprandial glucose level by -2.19 mmol/l (179). As per a 2 month long double blind placebo controlled trial on 25 subjects with type 2 diabetes mellitus, hydroalcoholic extract of fenugreek seed also decreased insulin resistance compared to control which was apprehended by an increase in insulin sensitivity percentage (112.9 ± 67% vs 92.2 ± 57%, respectively) and beta-cell secretion percentage (86.3 ± 32% vs 70.1 ± 52%, respectively) through HOMA-IR test (180).

## *Vitis vinifera* L.

*Vitis vinifera* L. (family: Vitaceae), known as ‘Grapevine’ or ‘Red grape’ is native to southern Europe and western Asia. However, it is cultivated worldwide which makes it the largest fruit crop in the world. It contains many active components in its seed and skin, including polyphenols, flavonoids, proanthocyanidins, anthocyanins, procyanidins, and resveratrol, a stilbene derivative (181). The kir6.2 channel is encoded by the KCNJ11 gene. It has been shown that congenital hyperinsulinism is caused by a mutation in this gene and has a significant role in the development of type-1 diabetes. Pterostilbene has promising inhibitory efficacy on both normal and mutant models of kir6.2 as an active component of *V. vinifera*. Again, quercetin, myricetin and resveratrol are three other most notable polyphenols found in red grapes to treat diabetic complications. Quercetin can demonstrate improved expression of adiponectin in white adipose tissue and blood concentration, despite inhibition of poly (ADP-ribose) polymerase γ expression followed by improved insulin sensitivity (182). Quercetin can also inhibit glucose uptake at the level of glucose transporters (GLUTs) (183). Besides, to treat hyperglycemia, Myricetin is also used as traditional medicine in northern Brazil. Myricetin can promote glucose uptake in the liver and soleus muscles as well as hepatic glycogen synthase (184). Myricetin can also improve insulin resistance in fructose chow-fed rat models (185). In addition, it can also halt advanced glycation end products in diabetic conditions (184). Furthermore, antihyperlipidemic and human pancreatic alpha-amylase inhibitions are a few other promising mechanisms by which myricetin can produce a significant antidiabetic effect (186). Resveratrol, a phytochemical from stilbene class of polyphenolic compounds isolated from red grape can also demonstrate strong antidiabetic activity. It can also protect against diabetic nephropathy while administration of resveratrol along with protective activities in renal dysfunction and oxidative stress (187). Resveratrol can effectively restore cellular homeostasis by activating the redox plasma membrane system, which functions as a compensatory mechanism in the cell to preserve redox status (184, 188). Besides, resveratrol administration to diabetic rats has resulted in decreased concentration of glycosylated hemoglobin (189). The antihyperglycemic efficacy of resveratrol, demonstrated in diabetic animals has been suggested to be due to the stimulatory activity on the transportation of intracellular glucose. The involvement of resveratrol can also promote glucose uptake in diabetic rat models (190). Improved expression of the insulin-dependent glucose transporter (GLUT4) was reported after ingestion of resveratrol in the study conducted on diabetic rat models (191, 192). Resveratrol was also reported to modulate the function of sirtuin-1, which ameliorates homeostasis of whole-body glucose and insulin sensitivity in diabetic rats (192). Moreover, in a 3 month long randomized controlled clinical trial on 62 subjects with type 2 diabetes mellitus (age range: 30-70 years), resveratrol supplementation exhibited antihyperglycemic action by lowering the value of hemoglobin A1c compared to the control (9.65 ± 1.54 vs 9.99 ± 1.50, respectively) (193). In another 45 days long randomized double-blinded placebo-controlled parallel clinical study on 66 subjects (age range: 20-65 years) with type 2 diabetes mellitus, 1 g daily resveratrol supplementation exhibited antihyperglycemic action by lowering the values of fasting blood glucose, hemoglobin A1c, insulin secretion, and insulin resistance compared to baseline (194). In another 30 days long randomized, double‐blind, crossover trial, resveratrol supplementation lowered the response of postprandial glucagon in obese type 2 diabetic patients (195). Quercetin also reduced fasting blood glucose and insulin secretion, as per a meta‐analysis of randomized controlled trials (196).

## *Zingiber officinale* Roscoe

*Zingiber officinale* Roscoe (family: Zingiberaceae) also known as ‘Ginger’ is a perennial plant with slender, brightly green, grassy leaves and yellowish green flowers which is grown in the tropics. Rhizome serves as the edible and medicinal part of the plant. Its administration to streptozotocin (STZ)-induced diabetic rats was found to reduce serum glucose, cholesterol and triacylglycerol levels significantly. Besides, raw ginger also exhibited effectivity in reversing diabetic proteinuria in diabetic rats (197). Several active constituents have been isolated from Ginger including gingerols and their related dehydration products, the shogaols, as well as volatile oils including sesquiterpenes, such as β-bisabolene and monoterpenes, mainly geranial and neral (197). A previous study revealed that 6-shogaol and 6-gingerol can suppress the development of diabetic complications as well as advanced glycation end products (AGEs) by arresting methylglyoxal, the precursor of AGEs (198). 6-gingerol can also arrest Nϵ-carboxymethyl-lysine (CML), a marker of AGEs through activation of Nrf2 (199). 6-paradol and 6-shogaol facilitated glucose consumption by increasing AMPK phosphorylation in 3T3-L1 adipocytes and C2C12 myotubes. Furthermore, 6-paradol considerably decreased the concentration of blood glucose in high-fat diet-fed mouse models (200). Besides, 6-gingerol, in type 2 diabetic mice, aided glucose-stimulated insulin secretion and improved glucose tolerance by upraising glucagon-like peptide 1 (GLP-1). In addition, 6-gingerol therapy galvanized glycogen synthase 1 and increased glucose transporter type 4 (GLUT-4) cell membrane presentations which amplified skeletal muscles’ glycogen storage (201). A meta-analysis study on 10 randomized-controlled clinical trials conducted on 490 type 2 diabetic subjects revealed that ginger improves glycemic control by lowering fasting blood glucose and HbA1c levels. Insulin sensitivity was also improved. Lipid profile improved as well (202). Moreover, the supplementation of ginger improved the recovery of inflammation in type 2 diabetic patients in a randomized-controlled clinical trial, suggesting that the supplementation could help lessen diabetes associated chronic complications (203).

## Other Promising Plants and Phytocompounds

Researchers suggested that there are almost 800 dietary and non-dietary plants with antidiabetic properties (77). So it is not possible to describe every plant and its isolated bioactive phytochemicals in a single study. However, a few more notable antidiabetic plants along with their phytochemicals and possible antidiabetic actions are summerized in **Table 1** in addition to the aforementioned plants.

**Table 1:** Plants with antidiabetic properties along with responsible phytochemicals and clinical trial studies.

Sl. No.	Name of the plant	Family	Compounds	*In vitro/In vivo* study with mechanism of action	Clinical Trials				References
					Description	Dosage	Participants	Outcomes	
1	*Allium sativum* L.	Amaryllidaceae	Allicin, Alliin, Diallyl trisulfide, S-allyl cysteine, Allyl mercaptan, Ajoene	Inhibition of DPP-4 enzyme	Nine randomized-controlled trials on with a duration of 1 to 2 weeks	0.05g to 1.5g dose of garlic	768 type 2 diabetic patients	Reduction of fasting blood glucose level, with a further reduction in glycosylated hemoglobin and fructosamine in 12 to 24 weeks	(204–206)
2	*Aegle marmelos* Correa.	Rutaceae	Aegeline	Elevation of blood insulin levels along with liver glycogen	Leaf juice supplementation	20 g/100 ml dose for 4 weeks	60 type 2 diabetic patient (25 were male and 35 were female; age range: 25-69 years old)	Reduction in fasting blood glucose, glycosylated hemoglobin (by 20%) and postprandial blood glucose (by 31%)	(207)
3	*Artocarpus heterophyllus* Lam.	Moraceae	Gallic acid, Catechin, Caffeic acid, Rutin and Quercetin	Inhibits α-glucosidase activity in a dose-dependent manner, increase liver glycogen,increases glucose transporter 2 concentration, reduces blood glucose level	6 month long randomized double blind placebo controlled cross over trial	Mixed herbal preparation (*Artocarpus heterophyllus* + *Salacia reticulata* + *Cinnamomum zeylonicum* + *Pterocarpus marsupium*)	51 type 2 diabetic patients	HbA1C value and the dose of glibenclamide was significantly lowered.	(208–210)
4	*Bauhinia forficate* Link	Fabaceae	Kaempferitrin	Exhibits hypoglycemic effect in normal and in alloxan-induced diabetic rats, reduces plasma and urinary glucose level in streptozotocin-diabetic rats	3 month long quasi-experimental pilot study	0.4% *B. forficata* tea in 200 mL water twice daily	25 type 2 diabetic patients	0.25% reduction in HbA1C; amelioration in lipid profile	(211–213)
					3 month long pilot clinical study	0.15% infusion of *B. forficata* thrice daily	15 prediabetic and diabetic volunteers	0.57 +/- 0.83% reduction in HbA1C
					75 day long clinical trial	Infusion of *B. forficata* leaves	20 type 2 diabetic patients	Significantly reduced glycemic profile	(214)
5	*Beta vulgaris* L.	Chenopodiaceae	Betavulgarosides (II, III, IV), Apigenin 8-C-β-D-glucopyranoside (vitexin), Acacetin 8-C-β-D-glucopyranoside, Acacetin 8-C-α-L-rhamnoside	Reduces blood glucose level, inhibits α-glucosidase activity	6 week long clinical trial	Daily dose of 10% *B. vulgaris* juice	30 healthy volunteers	Significant reduction of plasma glucose; down-regulation of insulin and C-peptide along with increase in cortisol levels	(215–217)
6	*Bougainvillea spectabilis* Willd.	Nyctaginaceae	D-pinitol	Increase in the activity of glucose-6-phosphate dehydrogenase and hepatic, skeletal muscle glycogen content	Randomized-controlled clinical study	1.2 g dose	66 type 2 diabetic patients (20 were male and 46 were female; age range: 20-75 years)	Reduction in HbA1c, HOMA-IR and fasting blood glucose levels	(218–221)
					Randomized parallel single-blind placebo and cross-over-controlled trial	6.0 g dose	30 healthy subjects (11 were male and 19 were female; age range: 18–65 years)	Reduction in blood glucose and insulin levels by 45 and 60 minutes, respectively	(222)
					Randomized, double-blind, placebo-controlled, crossover trial	0.6g dose	20 healthy subjects (12 were male and 8 were female; age range: 18-25 years)	Reduction in post-prandial blood glucose levels	(223)
7	*Cecropia obtusifolia* Bertol.	Urticaceae	Isoorientin, Chlorogenic acid	Decreases HbA1c, reduces blood glucose level	21 day long double blind, randomized-controlled trial	Dry leaf infusion of *C. obtusifolia*	43 type 2 diabetic patients	15.25% reduction in fasting blood glucose; ameliorated lipid profile	(224–226)
					32 week long intervention	Aqueous leaf extract of *C. obtusifolia*	12 type 2 diabetic patients	Blood glucose and HbA_1c_ levels reduced significantly
8	*Centella asiatica* (L.) Urb.	Apiaceae	Asiaticoside (triterpene saponin compound), MadecassicAcid, Asiatic acid, Brahmoside and Brahminoside (glycosides)	Increase secretion of insulin from pancreatic β-cells, asiatic acid protects pancreatic β-cells from death *via* activation of Akt kinase and Bcl-xL, promotes proliferation of pancreatic β-cells, reduce blood glucose level and increase serum insulin level	6 month long randomized controlled trial	60 mg twice daily dose of total triterpenic fraction of *C. asiatica*	50 patients with diabetic microangiopathy	Decreased rate of swelling in ankles, lessened edema, venoarteriolar response and resting flux	(227–231)
					Prospective randomized control trial	Thrice daily dose of 2 capsules, each containing 50 mg freeze dry lyophilized extract of *C. asiatica*	200 diabetic patients with foot ulcers	Shortened duration of would healing, better wound contraction, reduced scar formation
					14 month long prospective, interventional, controlled trial	30 mg daily dose of the triterpene fraction of *C. asiatica* combined with *Melilotus* and other flavonoids	40 type 2 diabetic subjects with cystoid macular edema unaccompanied by macular thickening	Improvement of the sensitivity of retina; Disappearance of intraretinal cysts
9	*Lagerstroemia speciosa* (L.) Pers.	Lythraceae	Corosolic acid (2α-hydroxyursoloic acid)	Glucose transport-stimulating activity, stimulate glucose uptake in 3T3-L1 cells, reduce blood glucose level	2 week long randomized clinical trial	32 and 48 mg daily dose of *L. speciosa* leaf extract standardized to 1% corosolic acid in soft and hard gel formulation	10 type 2 diabetic patients	30% and 20% reduction in blood glucose level for soft and hard gel formulation, respectively	(232–234)
					Clinical study	A combination of the aqueous extract of *L. speciosa, Garcinia*, green coffee and green tea	24 mild type 2 diabetic patients	Blood glucose level reduced by 13.5% on average
					1 year long open label trial	100 mg tablet daily containing water soluble *L. speciosa* extract	15 type 2 diabetic patients	Fasting plasma glucose reduced by 16.6%, ameliorated glycated albumin and glucose tolerance
					2 week long clinical trial	Daily dose of 10 mg corosolic acid as a *L. speciosa* extract standardized to 18% corosolic acid in the form of soft gel capsule	12 non diabetic subjects	12% reduction in fasting blood glucose and post-prandial blood glucose
					Double blind cross-over clinical trial	Intake of 10 mg corosolic acid containing capsules followed by 75 g oral glucose tolerance test 5 minutes later.	31 subjects	Lessened blood glucose levels during 60-120 minutes, corosolic acid is responsible for the blood glucose level lessening activity
10	*Laminaria japonica* Aresch.	Laminariaceae	Butyl‐isobutyl‐phthalate, polysachharides	Inhibits the activity of α-glucosidase, reduces fasting blood glucose level, increases plasma insulin level	12 week long double-blind randomized placebocontrolled trial	4 capsules daily containing 350 mg of *L. japonica* extract	37 type 2 diabetic patients	FBG and HbA1c values were not reduced significantly, lipid profile was significantly lowered, antioxidant status was ameliorated	(235–238)
11	*Mangifera indica* L.	Anacardiaceae	Mangiferin, Kaempferol	Imporves oral glucose tolerance, reduces fasting plasma glucose level, inhibits the activity of α-amylase and α-glycosidase	3 month long randomized controlled trial	5 g of powdered mango leaves daily	50 type 2 diabetic patients	Lessened blood glucose levels, reduced weight, symptomatic relief	(239–244)
					12 week long pilot study	10 g ground freeze-dried mango pulp daily	20 obese adults	Blood glucose significantly lessened
					12 week long double-blind randomized controlled trial	150 g daily dose of mangiferin	97 overweight patients	Significant reduction in insulin resistance index, lipid profile was ameliorated by reducing TG and FFA levels
					42 day long clinical study	400g of mango pulp daily	21 healthy lean and obese subjects	Long term glucose hemostasis is achieved in obese subjects
12	*Salacia chinensis* L.	Celastraceae	Salasones A, B, and C, Salaquinone A, Salasol A, 22-dihydroxyolean-12-en-29-oic acid,Tingenone, Tingenine B, Regeol A,Triptocalline A	Inhibits α-glucosidase activity, inhibits rat lens aldose reductase	Randomized, double-blind, placebo-controlled, cross-over trial	1000 mg hydroalcoholic extract of stems and roots of *S. chinensis*	30 healthy adult volunteers	Reduction in post-prandial blood glucose levels	(245–251)
					Randomized double-blind, placebo controlled, crossover trial	Various doses of the extract of *S. chinensis* (200 mg, 300 mg, and 500 mg)	35 healthy subjects	Ameliorated postprandial glucose level and insulin response	
					Double blind randomized controlled trial	Either 300 mg or 500 mg dose of *S. chinensis* extract	48 healthy overweight or obese participants	Reduced glycemic indices supporting it’s α-GI activity, gastrointestinal peptides were affected which might lead to apetite modification	
					Double-blind randomized, placebo-controlled crossover trial	150, 300, or 600 mg of *S. chinensis* extract	Diabetic patients	Postprandial blood glucose and insulin levels were suppressed significantly and dose-dependently	
					12 week double-blind randomized, placebo-controlled parallel group trial	600 mg daily dose of *S. chinensis* extract	Diabetic patients	HbA1c level and glucose tolerance were ameliorated significantly	
					Pilot study	Twice daily dose of 1000 mg of *S. chinensis*	30 stable diabetic patients with chronic kidney disease	Renoprotective role was observed through reduction in IL-6 and homocysteine levels	
13	*Salacia reticulata* Wight	Celastraceae	Salacinol, Kotalanol	Inhibits α-glucosidase enzyme	6 month long randomized double blind placebo controlled cross over trial	Mixed herbal preparation (*Salacia reticulata* + *Artocarpus heterophyllus* + *Cinnamomum zeylonicum* + *Pterocarpus marsupium*)	51 type 2 diabetic patients	HbA1C value and the dose of glibenclamide was significantly lowered.	(210, 252–257)
					6 week long placebo-controlled, cross-over trial	240 mg daily dose of *S. reticulata* extract	20 type 2 diabetic subjects	Lessened fasting blood glucose, glycated hemoglobin levels and BMI value	
					60 day long clinical study	2 g of powdered *S. reticulata* bark daily	40 type 2 diabetic patients	Significant reduction of blood glucose, Hb1Ac levels and improvement of lipid profile	
					6 week long double-blind, placebo-controlled, randomized trial	500 mg *S. reticulata* root bark and leaf extract daily	29 prediabetic participants	Both leaf and bark extract showed FBG lowering activity after 6 weeks	
14	*Scoparia dulcis* L.	Scrophulariaceae	Scutellarein, Apigenin, Luteolin, Scopadulcic acid B, Betulinic acid, Scoparic acid A	Inhibition of α-glucosidase activity, stimulateS the beta cell to secrete insulin., ameliorated glucose uptake activity, exhibits PPAR-γ agonistic activity and increase insulin sensitivity	6 month long randomized crossover clinical trial	1 packet of *S. dulcis* leaf extract made porridge once daily and thrice weekly	35 subjects with type 2 diabetes	Reduced fasting blood glucose and Hb1Ac level	(258, 259)
15	*Stevia rebaudiana* Bertoni	Asteraceae	Stevioside	Antihyperglycaemic, insulinotropic and glucagonostatic actions	60 day long clinical study	1 g *S. rebaudiana* leaf powder	20 type 2 diabetic patients	Reduced fasting and post-prandial blood glucose levels, ameliorated lipid profile	(260–264)
					45 day long clinical trial	Thrice daily dose of 0.5g and 1g of powdered leaves of *S. rebaudiana* with tea	15 diabetic patients	Reduction in FBG and PPBG, although statistically insignificant	
					Acute, paired cross-over clinical trial	1 g Stevioside	12 type 2 diabetic patients	Reduced postprandial blood glucose levels, 40% increment in insulinogenic index, AUC of glucose response curve decreased by 18%	
					Study assessing low calorie sweeteners	400 g (250 kcal) preload sweetened with *S. rebaudiana*	12 healthy obese and 19 lean subjects	Significant reduction in insulin and PPBG levels	

## Molecular Mechanisms of Medicinal Plants and/or Extracted Phytochemicals to Treat Diabetes Mellitus

### a. Inhibition of α Glucosidase Secreted From Brush Border of the Small Intestine

Mammalian α glucosidase, a membrane-bound hydrolytic enzyme found in the mucosal brush border of the epithelia of the small intestine plays a key role in carbohydrate digestion. Inhibitors of this α glucosidase enzymes delay the cleavage of carbohydrates resulting in reduced glucose absorption and an attenuated postprandial glycemic level. Thus, α glucosidase inhibitors could show a beneficial effect in the management of non-insulin-dependent diabetes mellitus (NIDDM) by causing a reduction in postprandial blood glucose levels (265, 266).

### b. Inhibition of DPP-4 Enzyme

Glucagon like peptide-1 (GLP-1) and Glucose dependent insulinotropic polypeptide (GIP) are incretin hormones which can initiate the differentiation of β-cells, stimulate the biosynthesis and secretion of insulin and inhibit gastric emptying. However, these hormones are rapidly broken down by a serine peptidase enzyme known as dipeptidyl peptidase-4 (DPP-4). Therefore, inhibitors of DPP-4 can be used in the treatment of type 2 diabetes. These DPP-4 inhibitors exhibit their antidiabetic activity *via* prolongation of GLP-1 and GIP activity, stimulation of insulin release and inhibition of glucagon secretion which ultimately leads to regulation of the blood glucose level (267, 268).

### c. Inhibition of α Amylase Secreted From Salivary Gland

Hydrolysis of α-1,4-glucan polysaccharides, such as starch and glycogen is carried out by the enzyme, α-amylase which is found mainly in the saliva and pancreatic juice. Inhibition of this enzyme helps in the prevention of high postprandial blood glucose levels (269).

### d. Increased Insulin Secretion

Past studies have shown that an increase in intracellular calcium ion [Ca2+]i was associated with insulin secretion. Generally, the release of insulin from the vesicles of the pancreatic β-cells is stimulated by a rise in [Ca2+]i. Membrane depolarization caused by the closure of the ATP-sensitive K+ channels of the insulin secreting β-cells leads to activation and opening of the voltage-dependent Ca2+ channels which increases the [Ca2+]i. These increased intracellular calcium levels stimulate the secretion of insulin from pancreatic β-cells. However, a few phytochemicals, for example, p-methoxycinnamic acid, has shown to increase insulin release by acting on the L-type Ca2+ channels rather than the ATP-sensitive K+ channels. This may also lead to a rise in cAMP *via* the inhibition of phosphodiesterase (270, 271).

### e. Increased Insulin Sensitivity and Enhanced Glucose Uptake by Muscle Cells and Adipose Tissue

Some phytochemicals improve the sensitivity of non-pancreatic cells to insulin resulting in improved glycemic control. In skeletal muscle and adipose tissue, glucose uptake is enhanced *via* the activation of a series of events which take place following an increase in insulin levels. When insulin binds to the insulin receptors, it causes phosphorylation of protein substrates leading to the activation of phosphatidylinositol 3-kinase (PI3K) and downstream signaling through PKB/Akt and PKC-λ/ζ. As a result, GLUT4, insulin-regulated glucose transporter protein is recruited to the cell membrane and an increase in the uptake of circulating glucose by the muscle cells and adipose tissue occurs *via* facilitated diffusion through GLUT4 transporter protein (272).

### f. Nourishment of Pancreatic β-Cells

Survival, restoration and maintenance of the mass/function of pancreatic β-cells can hinder the pathogenesis of diabetes mellitus. β-cells from the pancreas secrete the hormone insulin which is crucially salient in maintaining homeostasis for glucose metabolism in the body. β-cells are impaired in type 1 diabetes due to macrophage, cytokine and T-cell mediated autoimmune reactions. In the case of type 2 diabetes, β-cells could possibly be debilitated or rendered dysfunctional due to factors like oxidative stress, enduringly elevated glucose or lipid levels, and the release of the inflammatory mediators. To inhibit such destruction, β-cells can be fortified against reactive oxygen species (ROS) accretion and lipid peroxidation mediated cell death by augmenting both non-enzymatic (e.g., reduced glutathione) and enzymatic (e.g., superoxide dismutase, glutathione S transferase, glutathione peroxidase, catalase) antioxidants which can enhance the antioxidant capacity of the cell. In addition, increment of the secretion of β-cell anti-apoptotic genes (e.g., Bcl-2 proteins) and minimizing the secretion of pro-apoptotic genes (e.g., caspases) hinders DNA and subsequent cell damage. Inhibition of the pro-inflammatory transcription factor NF-κB reduces the inflammation stimulated production of inducible nitric oxide synthase (iNOS) and NO, hence reducing cell damage through increasing Ca2+ level in ER and mitigating ER stress, inactivating the JNK pathway and thus actuating the PI3K/Akt signaling which supports cell proliferation, survival and growth. Suppression of the deterioration of β-cell through these mechanisms halts reduced insulin secretion, thereby avoiding the state of hyperglycemia (273, 274).

### g. Reduction of HbA1c and Glycated Plasma Protein Concentration

Since diabetes mellitus is a condition where blood carbohydrate concentration increases, the monosaccharides nonenzymatically react with the proteins in blood (mainly hemoglobin A and albumin) and adheres to form a modified protein complex (Schiff base) through a process called glycation. The produced glycated hemoglobin (HbA1c) and glycated plasma proteins are concerned with much significance in the research. HbA1c value is often examined as it is one of the major markers for the diagnosis of diabetes mellitus. These glycated products can further encounter intramolecular rearrangements followed by other irreversible reactions (condensation, cross-linking, glycoxidation, cyclization, dehydration, etc.) to become advanced glycation end products (AGEs) which can accumulate and cause deleterious effects on metabolic and vascular health, leading to added diabetic complications. Glycation inhibitors hinder this process through various mechanisms, namely competitively binding with the amino group of the protein, binding at the site of glycation, cutting the open chain structure of monosaccharides and, adhering to the intermediaries of the glycation reaction. Hence, the concentration of HbA1c and glycated plasma proteins is lessened and the aftermath of glycation and diabetic complications can be avoided (275–277).

### h. Enhancement of GLP-1

Glucagon-like peptide-1 (GLP-1) is a hormone secreted by the L cells in the distal ileum and colon of the gastrointestinal system. Secreted upon nutrient intake, GLP-1 subsequently binds to GLP-1 receptor (a G-protein-coupled receptor) on pancreatic β-cell to exert its effects, namely, raising the glucose-dependent secretion of insulin and lessening the secretion of glucagon, decelerating gastric emptying, subduing of appetite with imparting a feeling of fullness. Circulatory GLP-1 faces immediate degradation by the enzyme dipeptidyl peptidase 4 (DPP-4) and hence has a short half life of about 2 min. As such, alternative compounds with the functionality of serving as agonists to GLP-1 receptor (GLP-1R) and being resistant to degradation by DPP-4 enzyme have been recognized feasible to employ the proper effect of GLP-1. The GLP-1 receptor (GLP-1R) agonists enhance insulin biosynthesis by increasing the transcription of the insulin genes through activating cAMP/PKA-dependent and -independent signaling mechanisms, increasing Ca2+ levels intracellularly, and activating the insulin gene promoting transcription factor pancreas duodenum homeobox 1 (Pdx-1). The agonist binding also results in inhibition of ATP-sensitive K+ channels, leading to depolarization of the membrane and simultaneous influx of extracellular Ca2+. ATP synthesis is also enhanced in mitochondria. The combined effect of surged ATP and intracellular Ca2+ level is the exocytosis of insulin storage granule. As a result, insulin secretion capability and reserve of β-cell is maintained. Glucagon secretion is inhibited by GLP-1R agonists either directly by acting on α-cells of the pancreas, or less likely indirectly along with the stimulated secretion of insulin. This inhibition ameliorates glycemic control as reduced glucagon level diminishes glucose production from the liver, which in turn reduces the required insulin in the bloodstream. Enhancement of GLP-1 also facilitates the indirect suppression of gastric emptying through the vagus nerve and its involvement with the central nervous system (CNS) located GLP-1Rs, thus relaying a sensory message to the brainstem (278, 279).

### i. Regulation of GLUT-4

Belonging to the group of sugar transporter proteins (GLUT1-GLUT12, and HMIT), glucose transporter type 4 (GLUT-4) is a 12-transmembrane domain containing transporter which allows insulin induced blood glucose influx into skeletal muscle and fat cells through facilitated diffusion process and hence maintains the homeostasis of glucose metabolism in the body. The transporter typically resides intracellularly but relocates to the cell membrane upon stimulation of insulin or during exercise through independent mechanisms. The receptor binding of insulin in target cells activates insulin receptor (IR) tyrosine kinase, thus beginning phoshphorylation of tyrosine moiety of insulin receptor substrate proteins (IRS) followed by phosphoinositide 3-kinase (PI3K) recruitment. Afterward, PI3K catalyzed phosphorylation of phosphatidylinositol-4, 5-bisphosphate (PIP2) produces phosphatidylinositol-3, 4, 5-triphosphate (PIP3), which in turn triggers the phosphorylation mediated activation of other protein kinases (Akt, aPKCλ/ζ) that eventually mobilize the effectors, namely Rab proteins. Rab proteins (Rab8 and Rab14) lead to GLUT-4 translocation into the cell membrane from intracellular GLUT4 storage vesicle (GSV) which increases glucose internalization up to 10-20 times (280, 281).

## Chemical Class Wise Few Most Prominent Antidiabetic Phytochemicals Along With the Reported Mechanism of Actions

Based on previous research studies, few phytochemicals have been already recognized as the most prominent antidiabetic lead compounds. Those are currently under exclusive assessment so that novel antidiabetic drugs can be introduced in the coming days. In **Tables 2**–**5** the few most prominent phytochemicals with the reported mechanism of actions are represented briefly. Alongside these phytochemicals, concerned researchers should also evaluate other aforementioned phytochemicals in this review work to establish absolute safety and toxicity profile as well as the mechanism of antidiabetic actions.

**Table 2:** Antidiabetic potential of alkaloids extracted from medicinal plants and their mechanism of actions.

Sl. No	Compounds	Plant source	Study model	Mechanism of action	Reference
1	Aegeline	*Aegle marmelos* Correa	*In vivo* (Diabetic rat)	Lowering of blood glucose level due to similarity in structure and action with b3-AR agonists	(207)
2	Berberine	*Berberis aristata* DC.	*In vivo* (Diabetic rat)	Improving the action of insulin by triggering AMPK; reducing insulin resistance through protein kinase C-dependent up-regulation of insulin receptor expression; causing glycolysis; enhancing GLP-1 secretion and regulating its release, inhibiting DPP-IV	(30)
3	Vindoline, Vindolidine, Vindolicine, Vindolinine	*Catharanthus roseus* (L.) G.Don	*In vitro*	Vindoline, vindolidine, vindolicine and vindolinine induced increased glucose uptake in myoblast C2C12 or pancreatic β-TC6 cells. Vindolicine, vindolidine and vindolinine also improved protein tyrosine phosphatase-1B (PTP-1B) inhibitory functions	(45)
4	Cryptolepine	*Cryptolepis sanguinolenta* (Lindl.) Schltr.	*In vivo* (Diabetic mouse)	Enhanced glucose transport	(78)
5	Radicamines A, Radicamines B	*Lobelia chinensis* Lour.	*In vitro*	Inhibition of α-glucosidase activity	(103)
6	Lupanine, 13-a-OH lupanine, 17-oxo-lupanine	*Lupinus perennis* L.	*In vivo* (Diabetic rat)	Enhanced the secretion of insulin in a glucose-dependent manner by reducing K+ permeability in the β-cell plasma membrane	(106)
7	Moringinine	*Moringa oleifera* Lam.	*In vivo* (Diabetic rat)	Aiding the restoration of the normal histological structure of the pancreas	(122)
8	1-deoxynojirimycin	*Morus alba* L.	*In vivo* (Diabetic mouse)	Reduction in the activity of α-glucosidase by competitive inhibition	(130)
9	Nuciferine	*Nelumbo nucifera* Gaertn.	*In vitro*	Increase in insulin secretion in both isolated islets and INS-1E cells, stimulation of both the first phase and the second phase of insulin secretion by closing K-ATP channels and also through stimulation of K-ATP channel independent amplification pathways.	(137)
10	Gentianine	*Swertia chirayita* Buch Ham.	*In vitro*	Promising amelioration in adipogenesis associated expression of PPAR-γ, GLUT-4 and adiponectin	(164)
11	Magnoflorine	*Tinospora cordifolia* (Willd.) Miers	*In vitro*	Potent inhibition of α-glucosidase	(172)

**Table 3:** Antidiabetic potential of phenolics extracted from medicinal plants and their mechanism of action.

Sl. No	Compounds	Subclass	Plant source	Study model	Mechanism of action	Reference
1	Piceatannol	Stillbenes	*Callistemon rigidus* R.Br.	*In vivo* (Diabetic mouse)	Suppression in the activity of α- amylase.	(44)
2	Scirpusin B	Stillbenes	*Callistemon rigidus* R.Br.	*In vivo* (Diabetic mouse)	Regulation of α-amylase in mouse GIT. Suppression in the activity of α- amylase.	(44)
3	Chamaemeloside	Flavonoids	*Chamaemelum nobile* (L.) All.	*In vivo* (Diabetic mouse)	Potential suppression in the production of hepatic glucose, as such, reduced gluconeogenesis. Potential effects on intestinal absorption, heptic or peripheral disposal of glucose as well	(49)
4	Pyrogallol	Phenols	*Cinnamomum verum* J. Presl	*In vivo* (Diabetic mouse)	Renovation of beta cells	(56)
5	Bisdemethoxycurcumin, Curcumin, Demethoxycurcumin	Phenols	*Curcuma longa* L.	*In vitro*	Inhibition of α-glucosidase activity	(71)
6	Acacetin	Flavonoids	*Lobelia chinensis* Lour.	Network pharmacological model	Promotion of secretion of insulin, improvement of insulin resistance, and stimulation of the utilization of glucose by acting on GSK3B, MAPK, INR, and dipeptidyl peptidase-4 (DPP4)	(104)
7	Coumarins	Coumarins	*Aegle marmelos* Correa	*In vivo* (Diabetic rat)	Stimulation of insulin secretion from beta cells of the isles of Langerhans	(282)
8	Quercetin	Flavonoids	*Matricaria chamomilla* L.	*In vivo* (Diabetic rat)	Quercetin moderately inhibited the enzymatic activity of sucrase	(111)
				Human	Halted sorbitol from accumulating in erythrocytes	
			*Moringa oleifera* Lam.	*In vivo* (Diabetic rat)	Aiding the restoration of the normal histological structure of the pancreas	(122)
				*In vivo* (Diabetic rat)	Blocking the transport of fructose and glucose by GLUT2 in the brain and promoting the translocation and expression of GLUT4 in skeletal muscle	(125)
			*Vitis vinifera* L.	*In vivo* (Diabetic rat)	Improvement of the expression of adiponectin in white adipose tissue and blood concentration, in spite of an inhibition of poly (ADP-ribose) polymerase γ expression followed by improved insulin sensitivity. Inhibition of glucose uptake at glucose transporters (GLUTs) level	(182, 183)
9	Luteolin	Flavonoids	*Matricaria chamomilla* L.	Human	Halted sorbitol from accumulating in erythrocytes	(111)
10	Esculetin, Umbelliferone	Coumarins	*Matricaria chamomilla* L.	*In vivo* (Diabetic rat)	Esculetin showed moderate inhibition in the enzymatic activity of sucrase	(111)
				Human	Esculetin and umbelliferone halted sorbitol from accumulating in erythrocytes	
11	Isoquercitrin, Astragalin	Flavonoids	*Morus alba* L.	*In vivo* (Diabetic mouse)	Inhibition α-glucosidase activity	(130)
12	Valoneic acid dilactone	Tannins	*Punica granatum* L.	*In vivo* (Diabetic rat)	Inhibition of the activity of aldose reductase and protein tyrosine phosphatase 1B (PTP1B). Improvement in insulin secretion from pancreatic β cells or its release from the bound form along with insulin mimetic actions or amended glucose utilization technique	(150)
13	Karanjin	Flavonoids	*Pongamia pinnata* (L.) Pierre	*In vitro*	Inhibition of PTPase-1B	(156)
14	Pongamol	Phenols	*Pongamia pinnata* (L.) Pierre	*In vitro*	Inhibition of PTPase-1B	(156)
15	Silychristin A	Flavonolignan	*Silybum marianum* (L.) Gaertn	*In vivo* (Diabetic rat)	Improvement of the function of β-cells along with glucose lowering effect by protecting the β-cells from oxidative stress-induced damage and blocking the activity of α-glucosidase enzyme	(159)
16	Mangiferin	Xanthonoid	*Swertia chirayita* Buch Ham.	*In vivo* (Diabetic rat)	Exibition of glucosidase and 2,2-diphenyl-1-picrylhydrazyl radical inhibitory action	(165)
17	Pterostilbene	Stillbenoids	*Vitis vinifera* L.	Wild-type and mutant Kir6.2 models	Promising inhibitory efficacy on both normal and mutant models of kir6.2 channel which is encoded by the KCNJ11 gene, whose mutation causes congenital hyperinsulinism	(181)
18	Myricetin	Flavonoids	*Vitis vinifera* L.	*In vivo* (Diabetic rat)	Promotion of glucose uptake in liver and soleus muscles as well as hepatic glycogen synthase, halting advanced glycation end products in diabetic condition	(184)
				*In vivo* (Diabetic rat)	Improvement of insulin resistance	(185)
				*In vitro*	Human pancreatic alpha-amylase inhibition	(186)
19	Resveratrol	Stilbenoids	*Vitis vinifera* L.	*In vivo* (Diabetic rat)	Stimulation of the transportation activity of intracellular glucose and promotion of glucose uptake	(190)
				*In vivo* (Diabetic rat)	Improvement in the expression of insulin-dependent glucose transporter (GLUT4)	(191, 192)
				*In vivo* (Diabetic rat)	Modulation of the function of sirtuin-1, which ameliorates homeostasis of whole-body glucose and insulin sensitivity	(192)
20	6-shogaol	Phenols	*Zingiber officinale* Roscoe	*In vitro*	Suppression of the development of diabetic complicacies and advanced glycation end products (AGEs) by arresting methylglyoxal, the precursor of AGEs, arrest of Nϵ-carboxymethyl-lysine (CML), a marker of AGEs through activation of Nrf2.	(198, 199)
				*In vitro*	Facilitation of glucose consumption by increasing AMPK phosphorylation in 3T3-L1 adipocytes and C2C12 myotubes	(200)
21	6-gingerol	Phenols	*Zingiber officinale* Roscoe	*In vivo* (Diabetic mouse)	Aided glucose-stimulated insulin secretion and improved glucose tolerance by upraising glucagon-like peptide 1 (GLP-1). 6-gingerol also galvanized glycogen synthase 1 and increased glucose transporter type 4 (GLUT4) cell membrane presentations which amplified skeletal muscles’ glycogen storage	(201)
				*In vitro*	Suppressing the development of diabetic complications and advanced glycation end products (AGEs) by arresting methylglyoxal, the precursor of AGEs, arrest of Nϵ-carboxymethyl-lysine (CML), a marker of AGEs through activation of Nrf2.	(198, 199)
22	6-parodol	Phenols	*Zingiber officinale* Roscoe	*In vitro*	Facilitation of glucose consumption by increasing AMPK phosphorylation in 3T3-L1 adipocytes and C2C12 myotubes	(200)

**Table 4:** Antidiabetic potential of terpenes extracted from medicinal plants and their mechanism of actions.

Sl. No	Compounds	Subclass	Plant source	Study model	Mechanism of action	Reference
1	Bacosine	Triterpenoids	*Bacopa monnieri* (L.) Wettst.	*In vivo* (Diabetic rat)	Increase in the consumption of peripheral glucose and protection against oxidative damage. Increase in the level of liver glycogen as well	(29)
2	Bassic acid	Triterpene acid	*Bumelia sartorum* Mart.	*In vivo* (Diabetic rat)	Increase in glucose uptake and glycogen synthesis. Increase in insulin secretion from the pancreatic beta-cells	(42)
3	β-amyrin	Triterpenoids	*Costus pictus* D. Don	*In vitro*	Improved glucose uptake in 3T3-L1 adipocytes	(67)
4	Turmerone	Sesquiterpenoids	*Curcuma longa* L.	*In vitro*	Inhibition of α-glucosidase and α -amylase activity	(73)
5	α-amyrin-3O-β-(5-hydroxy) ferulic acid	Triterpenes	*Euclea undulate* Thunb. var. myrtina	*In vitro*	Inhibition of α –glucosidase activity	(81)
6	Gymnemagenin	Triterpenoids	*Gymnema sylvestre* R. Br.	Crystallographic investigation	Exhibition of good gelling property with various target protein’s crystallographic constitution which contribute to its carbohydrate management property	(83)
7	Thymoquinone, Dithymoquinone	Monoterpene, Diterpene	*Nigella sativa* L.		Potential stimulation in pancreatic β-cells causing insulin secretion, reduced hepatic gluconeogenesis, and induced insulin sensitivity in peripheral tissue	(139)

**Table 5:** Antidiabetic potential of other notablephyto compounds extracted from medicinal plants and their mechanism of actions.

Chemical Class	Compounds	Plant source	Study model	Mechanism of action	Reference
Phenylpropanoids	Chlorogenic acid	*Cichorium intybus* L.	*In vivo* (Diabetic rat)	Increased glucose uptake in L6 muscular cells, elevated insulin secretion from the INS-1E insulin-secreting cell line and rat islets of Langerhans.	(53)
		*Moringa oleifera* Lam.	*In vivo* (Diabetic rat)	Aiding the restoration of the normal histological structure of the pancreas	(122)
	Chicoric acid	*Cichorium intybus* L.	*In vivo* (Diabetic rat)	Increased glucose uptake in L6 muscular cells, elevated insulin secretion from the INS-1E insulin-secreting cell line and rat islets of Langerhans along with insulin secreting and sensitizing action	(53)
	Eugenol	*Cinnamomum verum* J. Presl	*In vivo* (Diabetic mouse)	Renovation of beta cells	(56)
	Cinnamaldehyde	*Cinnamomum verum* J. Presl	*In vivo* (Diabetic rat)	Ameliorating the uptake of glucose by upraising the amount of AKT2 and aortic nitric oxide synthase 3 (eNOS), insulin receptor substrate1 (IRS1) and p-85 regulatory subunit of PI3K (PI3K-P85) while concurrently abating the expression of NADPH oxidase 4 (NOX4)	(56)
Saponins	3-hydroxycucurbita-5, 24-dien-19-al-7, 23- di-O-β-glucopyranoside, Momordicine- II	*Momordica charantia* L.	*In vivo* (Diabetic mouse)	Promising insulin releasing property	(115)
Lipid	Methyl tetracosanate	*Costus pictus* D. Don	*In vivo* (Diabetic rat)	Improved glucose uptake in 3T3-L1 adipocytes	(68)
Fatty acid	Linoleic acid, Oleic acid	*Nigella sativa* L.	Human	Potential stimulation in pancreatic β-cells causing insulin secretion, reduced hepatic gluconeogenesis, and induced insulin sensitivity in peripheral tissue	(139)
Protein	Turmerin	*Curcuma longa* L.	*In vitro*	Inhibition of α-glucosidase and α -amylase activity	(73)
	Polypeptide-p	*Momordica charantia* L.	Human	Insulin mimicking activity	(115)
Carbohydrate	α-arabinose, α-xylose, α-glucose, α-rhamnose, α-mannose	*Morus alba* L.	*In vivo* (Diabetic rat)	Repair of pancreatic β-cells	(130)
	Peltalosa	*Psacalium peltatum* (Kunth) Cass.	*In vivo* (Diabetic mouse)	Potentially enhance secretion of insulin from the islets of Langerhans or increase utilization of glucose by peripheral tissues	(157).
Miscellaneous	Kinsenoside	*Anoectochilus roxburghii* (Wall.) Lindl.	*In vivo* (Diabetic rat)	Restoration of damaged pancreatic β cells, functionality against oxidative stress and NO factor, regulation of antioxidant enzymes and scavenging of free radicals	(28)
	D-pinitol	*Bougainvillea spectabilis* Willd.	*In vivo* (Diabetic mouse)	Exhibition of an insulin-like impact by acting through a post-receptor insulin action pathway, affecting the uptake of glucose	(283)
	5-hydroxymethylfural	*Lobelia chinensis* Lour.	Network Pharmacological model	Promotion of secretion of insulin, improvement of insulin resistance, and stimulation of the utilization of glucose by acting on GSK3B, MAPK, INR, and dipeptidyl peptidase-4 (DPP4)	(104)
	Neutral ginsenosides, Malonyl ginsenosides	*Panax ginseng* C. A. Meyer	*In vivo* (Diabetic rat)	Increase in insulin sensitivity	(143, 144)

## Future Research Directions

Diabetes is conventionally treated and managed by taking synthetic antidiabetic medications commercially available in the market. The major classes among these medicines are sulphonylureas (glibenclamide), biguanides (metformin), thiazolidinediones (pioglitazone), DPP-4 inhibitors (sitagliptin), alpha-glucosidase inhibitors (acarbose), glinides (repaglinide) and GLP-1 agonists (exenatide) (7, 284). Despite the mass prevalence and usage, the synthetic drugs accompany various side effects which include hypoglycemia (for sulphonylureas, glinides), weight gain (for sulphonylureas, thiazolidinediones), cardiovascular risk (for sulphonylureas, thiazolidinediones), pancreatitis (for DPP-4 inhibitors, GLP-1 agonists), hepatitis (for thiazolidinediones, DPP-4 inhibitors), cancer risk (for DPP-4 inhibitors, GLP-1 agonists), gastrointestinal effects (for biguanides, GLP-1 agonists), lactic acidosis (for biguanides) (285). These adversities and constraints associated with the prevailing synthetic medications entail the researchers to search for antidiabetic drugs from plant sources with a better safety and efficacy profile. Along the lines of many other diseases, diabetes mellitus has been treated with plant based medications for a long while owing to factors like marked efficacy, less toxicity and side effects, low cost, and availability (286, 287). An exclusive development of plant based drugs has seemingly occurred through evolutionary mechanisms, imparting the capability to interact with biomolecules (288, 289). Isolated phytochemicals are either used as drugs or availed as chemical leads or their analogs for synthesizing biologically active compounds. The prevalence of phytochemicals in the pharmaceutical scenario can be perceived by surveying all the authorized drugs registered globally within the time frame of 25 years before 2007, where roughly 50% of the drugs were majorly plant based natural products or their synthetic derivatives (285, 290). As an example, metformin, the extensively used drug in treating type 2 diabetes, is a drug derived from the plant *Galegine officinalis* (291). Antidiabetic action being one of the most popular fields of use of phytochemicals houses compounds from various chemical classes, namely flavonoids (quercetin), alkaloids (berberine), terpenes (thymoquinone), phenylpropanoids (chlorogenic acid) and others. The phytochemicals reported in this study revealed prominent antidiabetic action through various mechanisms like inhibiting α-glucosidase, α-amylase and DPP-4 enzyme, increasing insulin sensitivity and secretion, increasing glucose uptake by muscle cells and adipose tissue, nourishing pancreatic β-cells, etc. These various ways of phytocompounds to exert antidiabetic action illustrates the effectual diversity they can offer. Thus, potential phytocompound(s), isolated from medicinal plants or dietary materials, with proven preclinical and clinical antidiabetic efficacy can be the prospective and potential candidates for the development of novel antidiabetic drugs. For example, Charantin and Polypeptide-p isolated from *Momordica charantia* L. have been reported to exhibit potential antidiabetic activities in preclinical and clinical studies which have been included in this manuscript. These two compounds can be prospective candidates for the development of novel antidiabetic medicaments after confirming their toxicitiy and further clinical trials. Traditional medicinal approaches like Ayurveda, Unani and so on also utilized plant based remedies to treat diabetic illness. However, further research is necessary to disclose the absolute and exact mechanism of action of these compounds which would facilitate their outset as drugs or chemical leads. Indeed, serving as drugs or drug templates is not the only purpose of plant derived compounds, rather, they also guide in the recognition and revelation of complex and novel molecular pathways and targets involving the health condition (292). Hence, further research on these phytochemicals could enable the discovery of several targets for therapeutic intervention against diabetes mellitus. In addition, elucidation of the feasibility and toxicity profile despite the mentioned predating advantage as plant based products is also a salient research concern.

## Discussion

The two most pronounced variations of diabetes are Type 1 diabetes mellitus and Type 2 diabetes mellitus both of which result in a hyperglycemic state. Type 1 diabetes mellitus is an autoimmune illness typified by the demolition of pancreatic β-cells followed by dreadful insulin scarcity. On the other hand, Type 2 diabetes mellitus is more familiar and a major portion of diabetic patients (90 to 95%) are suffering from this dysfunction, which is categorized by peripheral insulin resistance and anomalies in the secretion of insulin (284). However, it is a non-communicable illness, experts are warning us about a figure of almost 438 million diabetic patients in 2030 (7) which considers the dreadfulness of this disease. In a broader sense, the causative factors of diabetes can reside in insulin resistance, abnormal insulin secretion and hepatic glucose synthesis along with impaired fat metabolism. Insulin resistance refers to the state where the efficacy of the insulin on target tissues becomes compromised particularly on adipocytes, hepatocytes and skeletal muscles (293) which leads to hyperglycemia by impairing utilization of glucose and increasing hepatic glucose output (294). Despite having a good number of commercially available antidiabetic drugs, the side effects delimit their unquestionable implications. In contrast, nutraceuticals and phytomedicines offer a low incidence of adverse effects that can be a fantastic alternative to regular drugs in combating diabetes and its related complications (7). Plant-derived medicaments have also been mentioned in various ethnic and traditional practices including the Indian, Koran, Chinese and Mexican cultures as well as in Western and Ayurvedic herbalism approaches which accredits their tremendous antidiabetic potential (7, 295). Hence, the reported aforementioned phytochemicals in this extensive review can be considered as very promising wellsprings to develop novel antidiabetic therapeutics to heal diabetes and related complications.

## Conclusion

A collection of anti-diabetic plants used in the treatment of diabetes mellitus has been reviewed in this article. Several shreds of scientific evidence have proved that those phytochemicals possess antihyperglycemic potentials and can be effectively implicated in the management of diabetic and metabolic complications avoiding notable side effects exerted by conventional drugs. Although dietary and non-dietary plants are always considered as promising avenues of remedies to treat different types of disease states, together with diabetes and others, many plants and plant-derived bioactive phytoconstituents have not yet been researched well. In order to explore and validate proper mechanistic pathways of pharmacological activities demonstrated by the reported antidiabetic phytochemicals, further investigations are warranted. In spite of considering plants and/or dietary plant materials as safe for intake, yet the prospective antidiabetic phytochemicals should also be evaluated for toxicity studies for the establishment of therapeutically effective and safe phytomedicines.

## Author Contributions

MMRS and SA conceptualized the study. SA, MNRC and TNS searched in the databases and collected articles. SA, TNS and MNRC wrote the manuscript. MAR, NIC, CZ, JX, EH, SAK, and INM critically revised the manuscript. SA, TNS and NIC edited the final manuscript as per review comments. MMRS and INM critically evaluated, revised the manuscript and supervised the project. All authors contributed to the article and approved the submitted version.

## Conflict of Interest

The authors declare that the study was conducted in the absence of any commercial or financial relationships that could be construed as a potential conflict of interest.

## Publisher’s Note

All claims expressed in this article are solely those of the authors and do not necessarily represent those of their affiliated organizations, or those of the publisher, the editors and the reviewers. Any product that may be evaluated in this article, or claim that may be made by its manufacturer, is not guaranteed or endorsed by the publisher.

## Glossary

**Table:**

ADA	American Diabetes Association
AGEs	Advanced glycation end-product
AMP	Adenosine monophosphate
AMPK	5′ adenosine monophosphate activated protein kinase
b3-AR	Beta-3 adrenergic receptor
BMI	Body mass index
CIE	Cichorium intybus
CML	Carboxymethyl-lysine
DCMM	Dichloromethane-methanol
DNJ	1-deoxynojirimycin
DPP-4	Dipeptidyl peptidase-4
eNOS	Endothelial nitric oxide synthase
FBS	Fetal bovine serum
FPG	Fasting Plasma Glucose
GDM	Gestational diabetes mellitus
GI	Glycemic index
Glc-6-Pase	Glucose-6-phosphatase
GLP-1	Glucagon-like peptide-1
GLUT-4	Glucose transporter-4
GSK3B	Glycogen synthase kinase 3B
HbA1c	Hemoglobin A1c
HDL-c	High-density lipoprotein cholesterol
HepG2	Hepatoma cell line
HMBA	2-hydroxy 4-methoxy benzoic acid
HMG	3-hydroxy-3-methylglutaric acid
HOMA-IR	Homeostatic model assessment insulin resistance
IL-6	Interleukin-6
INR	International normalized ratio
INS-1E	Rat insulinoma cell line INS-1E
IRS1	Insulin receptor substrate1
K-ATP	ATP-sensitive potassium channel
LC/MS	Liquid chromatography-mass spectrometry
LDL-c	Low-density lipoprotein cholesterol
MAPK	Mitogen-activated protein kinase
NADPH	Nicotinamide adenine dinucleotide phosphate
NMR	Nuclear magnetic resonance
NO	Nitric oxide
NOX4	Nicotinamide adenine dinucleotide phosphate oxidase 4
p-AKT	Phosphorylated Akt
PKC	Protein kinase C
PPAR	Peroxisome proliferator-activated receptor
PTP1B	Protein tyrosine phosphatase 1B
PX-407	Poloxamer-407
SGOT	Serum glutamic oxaloacetic transaminase
SGPT	Serum glutamic pyruvic transaminase
SLM	Solid lipid microparticles
STZ	Streptozotocin
TC	Total cholesterol
TG	Triglycerides
2HPP	2 hour postprandial glucose
[Ca2+]i	Calcium ion
ATP	Adenosine triphosphate
PI3K	Phosphatidylinositol 3-kinase
PKB	Proteine kinase B
GIP	Glucose dependent insulinotropic polypeptide
MAMs	mitochondria-associated membranes
mTORC2	Mammalian target of rapamycin complex 2
PKC-λ/ζ	Protein kinase C zeta/lambda
PTEN	Phosphatase and tensin homolog
PP2A	Protein phosphatase 2
GAD	Glutamic acid decarboxylase
CD4	Cluster of differentiation 4
CD8	Cluster of differentiation 8
